# Climatic niche conservatism shapes the macroevolution, extinction, and survivorship of crocodile-line archosaurs

**DOI:** 10.1126/sciadv.aeg2217

**Published:** 2026-10-21

**Authors:** Alfio Alessandro Chiarenza, Alexandra F. C. Howard, Grace L. Varnham, Alex Farnsworth, Paul J. Valdes, Philip D. Mannion

**Affiliations:** ^1^ Department of Earth Sciences, University College London, London WC1E 6BT, UK.; ^2^ Science Group, Natural History Museum London, London, UK.; ^3^ Department of Ecology and Conservation Biology, Texas A&M University, College Station, TX, USA.; ^4^ Department of Zoology, University of Cambridge, Cambridge, CB2 3EJ, UK.; ^5^ School of Geographical Sciences, University of Bristol, University Road, Bristol BS8 1RL, UK.

## Abstract

Crocodylians are iconic thermophiles. Over 250 million years, crocodile-line archosaurs endured hyperthermals, climatic and range shifts, and two mass extinctions. Whether this niche conservatism reflects a deep evolutionary constraint or recent adaptation to novel climates remains unclear. We integrate present-day occurrences with a time-scaled phylogeny of >400 fossil and extant species. Niche models calibrated on living crocodylians were projected across 245 million years and compared with paleophylogeographic reconstructions. Macroevolutionary model fitting shows that temperature and precipitation exhibit strong phylogenetic structure, best described by bounded Ornstein-Uhlenbeck dynamics, indicating long-term niche conservatism. Poleward expansions in warm intervals and contractions in cool phases reflect tracking of conserved optima rather than niche evolution. Unlike non-avian dinosaurs, low-latitude suitability persisted across all but the most extreme end-Cretaceous scenarios, with recovery expanding from tropical refugia. Thermal and precipitation regimes shifted near the Cenomanian/Turonian hyperthermal (∼93.9 Ma) and Eocene/Oligocene aridification transition (∼33.9 Ma), respectively. Climatic niche conservatism is therefore ancient in crocodile-line archosaurs, but not completely static.

## INTRODUCTION

Understanding how climatic niches evolve across geological time, whether bounded by intrinsic physiological limits or expandable through adaptation to novel environments, is a central goal of evolutionary biology (*1*–*6*) and is key to conservation (*2*, *7*, *8*). Lineages can expand into new ecological spaces through adaptation or retain their ancestral ecospace when intrinsic constraints limit the range of phenotypes that are viable. This latter pattern is manifested ecologically via phylogenetic niche conservatism (PNC), the tendency for lineages to maintain ancestral ecological tolerances across speciation events (*9*, *10*) over macroevolutionary timescales. Whether such conservatism reflects genuine intrinsic constraints, limits on the range of trait values physiologically or developmentally accessible to a lineage, or is instead produced by selection consistently favoring ancestral trait values across all descendant lineages remains an open question (*11*, *12*). Resolving this question has relevance for understanding how physiologically conservative clades might respond to major climatic perturbations (*13*). If niches are labile, lineages may change their climatic tolerances in response to novel selective regimes; if conservative, survival depends on geographic tracking of suitable habitats via dispersal, with extirpation (and ultimately extinction) the likely outcome when tracking fails (*14*). Testing PNC at deep timescales therefore requires reconstructions of multidimensional environmental niches in both living species and their fossil relatives so that long-term evolutionary dynamics can be traced through time.

Ecological niche models (ENMs) built from extant occurrences capture the realized niche of living taxa, but only the fossil record provides empirical evidence of how clades responded to environmental perturbations across geological time (*15*, *16*). This distinction is particularly consequential for groups whose crown diversity is severely reduced relative to their past richness, because living representatives may sample only a small portion of the clade’s historical diversity, and likely of their ancestral niche space. Fossils are therefore essential for characterizing deep-time niche dynamics and for distinguishing whether present-day climatic associations reflect long-term niche conservatism, repeated niche shifts, or the ecological filtering of formerly broader clade-level tolerances. Integrated paleontological, phylogenetic, and climatic data spanning evolutionary timescales are needed to test these alternatives directly (*1*, *17*–*19*), particularly in groups with deep fossil records that preserve responses to major climatic perturbations.

Extant crocodylians (alligators, caimans, gavials, and “true” crocodiles), circumtropically distributed ectotherms with narrow thermal tolerances (*20*, *21*), exemplify the conservation consequences of climatic niche conservatism: 11 of the 26 currently recognized living species (Table 1) are listed as threatened (*22*), and all members face increasing pressure from climatic and environmental change. Their broader clade, the crocodile-line archosaurs (Pseudosuchia; Fig. 1A), originated approximately 250 million years ago (Ma), near the split from their bird-line cousins (*23*). Pseudosuchians diversified into terrestrial, semi-aquatic, fully marine, gigantic (>10^3^ kg), and even herbivorous forms (*24*), and occupied a broad latitudinal range that extended to the poles (*21*, *25*) (Fig. 1B). Despite this past ecological and distributional breadth, present-day crocodylians are confined to warm, low-latitude non-marine environments, a pattern (*20*, *21*) whose deep evolutionary origins and dynamics across the total group remain incompletely characterized. Pseudosuchians span two of the big five mass extinctions and every major Mesozoic and Cenozoic climatic oscillation, making them a compelling system for quantifying deep-time climatic niche dynamics, testing whether present-day thermophily reflects an intrinsic physiological limit or the legacy of selective extinction that pruned broader tolerances. Prior work has established that crown crocodylian distributions are thermally constrained (*20*, *21*), but whether this conservatism extends across the full 250–million year (Myr) total-group history, including ecomorphologically disparate stem lineages whose thermal physiology is debated (*26*), and whether temperature and precipitation niches are governed by similar or decoupled macroevolutionary dynamics, has not been tested with fully quantitative, spatiotemporally explicit methods.

**Table 1.** GBIF references for modern crocodylian occurrences.

Species	GBIF species page	19/02/2025
*Alligator mississippiensis*	https://www.gbif.org/species/2441370	19/02/2025
*Alligator sinensis*	https://www.gbif.org/species/2441368	19/02/2025
*Caiman crocodilus*	https://www.gbif.org/species/8953936	19/02/2025
*Caiman latirostris*	https://www.gbif.org/species/5846511	19/02/2025
*Caiman yacare*	https://www.gbif.org/species/5220210	19/02/2025
*Crocodylus acutus*	https://www.gbif.org/species/2441329	19/02/2025
*Crocodylus halli*	https://www.gbif.org/species/10671642	19/02/2025
*Crocodylus intermedius*	https://www.gbif.org/species/2441344	19/02/2025
*Crocodylus johnstoni*	https://www.gbif.org/species/9354985	19/02/2025
*Crocodylus mindorensis*	https://www.gbif.org/species/2441327	19/02/2025
*Crocodylus moreletii*	https://www.gbif.org/species/2441352	19/02/2025
*Crocodylus niloticus*	https://www.gbif.org/species/2441341	19/02/2025
*Crocodylus novaeguineae*	https://www.gbif.org/species/2441326	19/02/2025
*Crocodylus palustris*	https://www.gbif.org/species/2441350	19/02/2025
*Crocodylus porosus*	https://www.gbif.org/species/2441333	19/02/2025
*Crocodylus rhombifer*	https://www.gbif.org/species/2441322	19/02/2025
*Crocodylus siamensis*	https://www.gbif.org/species/2441348	19/02/2025
*Crocodylus suchus*	https://www.gbif.org/species/7852834	19/02/2025
*Gavialis gangeticus*	https://www.gbif.org/species/2441360	19/02/2025
*Melanosuchus niger*	https://www.gbif.org/species/2441409	19/02/2025
*Mecistops cataphractus*	https://www.gbif.org/species/2441405	19/02/2025
*Osteolaemus tetraspis*	https://www.gbif.org/species/2441385	19/02/2025
*Paleosuchus palpebrosus*	https://www.gbif.org/species/2441396	19/02/2025
*Paleosuchus trigonatus*	https://www.gbif.org/species/2441394	19/02/2025
*Tomistoma schlegelii*	https://www.gbif.org/species/2441378	19/02/2025

**Fig. 1.**
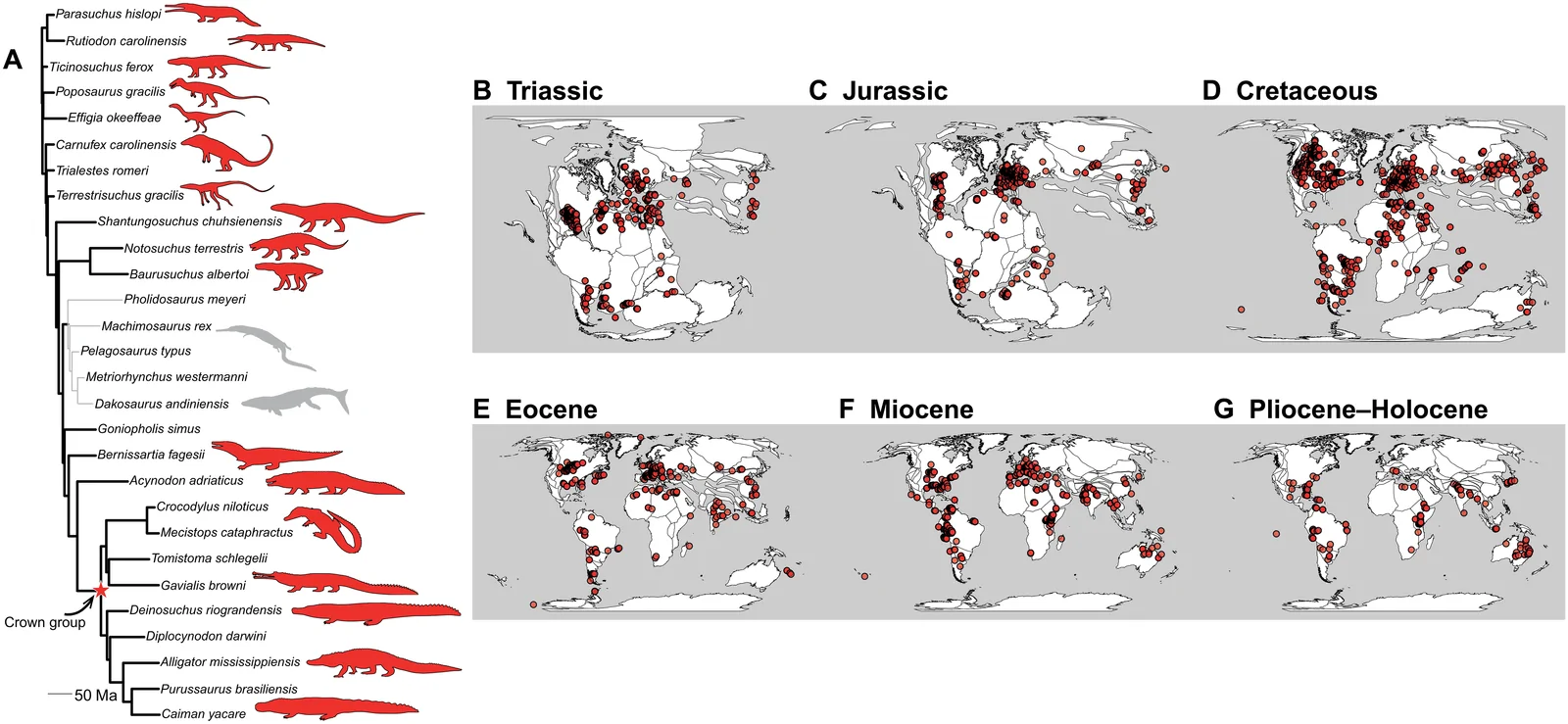
Evolution of Pseudosuchia in time and space. (**A**) Simplified phylogeny showing representative members of the 402 taxa used in this study (see Materials and Methods). Taxa in red are non-marine species included in the study; fully aquatic/marine species not used in this study are shown in gray. Red star indicates the clade Crocodylia. (**B** to **G**) Triassic–recent fossil record of Pseudosuchia plotted on paleogeographic maps based on plate models from Scotese (*177*) and Scotese *et al.* (*218*). Scale bar, 50 Ma (million years ago). Organism silhouettes are from PhyloPic (https://www.phylopic.org/; T. Michael Keesey, 2023) and were added using the rphylopic R package version 1.7.0 (*219*). Organism silhouettes are from PhyloPic (https://www.phylopic.org/; T. Michael Keesey, 2023) and were added using the rphylopic R package version 1.7.0 (*219*). Silhouettes were made by Edwin Price, 2023 (CC BY 4.0), David Stanley, 2024 (CC BY 4.0), Armin Reindl, 2021 (CC BY 4.0), Dan Folkes, 2025 (CC BY 4.0), Ananth Srinivas, 2025 (CC0 1.0), Scott Reid, 2021 (CC BY 3.0), Chase Brownstein, 2016 (CC BY 3.0), Slate Weasel, 2011 (Public Domain Mark 1.0), Jagged Fang Designs, 2021 (CC0 1.0), Miguel Hernández, 2026 (CC0 1.0), Smokeybjb, 2011 (CC BY 4.0), Nobu Tamura, 2011 (CC BY 4.0), Dmitry Bogdanov (vectorized by T. Michael Keesey), 2011 (CC BY 3.0), François-Louis Pelissier, 2023 (CC0 1.0), and An Ignorant Atheist, 2019 (CC0 1.0). Silhouettes were contributed by Edwin Price, Pascal Abel, Armin Reindl, Dan Folkes, Ananth Srinivas, Scott Reid, chasethedinosaur, Jagged Fang Designs, LiterallyMiguel, T. Michael Keesey, François-Louis Pelissier, and Jody Taylor.

Pseudosuchian diversity waxed and waned through the Mesozoic, with a major turnover across the end-Triassic mass extinction; surviving lineages radiated into novel ecomorphologies during the Jurassic and Cretaceous (*21*). After the Cretaceous/Paleogene (K/Pg) mass extinction (*27*, *28*), their Cenozoic record documents repeated poleward expansions during warm intervals [Paleocene-Eocene Thermal Maximum (PETM), Miocene Climatic Optimum] and contractions during cooling phases [Eocene/Oligocene (E/O) transition, Plio-Pleistocene glaciations] (*21*, *29*). This sequence of climatic pulses serves as a natural experiment that lets us track shifts in niche optima and their geographic consequences, quantify climatic trait evolution, and pinpoint episodes of selective extinction.

Here, we evaluate the pattern of climatic niche evolution across all non-marine pseudosuchians (Fig. 1A). To do so, we marshal an exceptionally broad dataset that spans deep-time and living diversity. Using a time-scaled phylogeny containing >400 taxa from >7000 fossil occurrences and all extant species (Fig. 1A), we overlay 130,000 vetted occurrence records for living crocodylians, collectively capturing their realized environmental space today and their past diversification. We generate ENMs for present-day crocodylians and project them onto 52 global paleoclimate reconstructions spanning the Anisian (Early Triassic, ∼245 Ma) to the present day, thereby estimating the climatic envelopes available to their terrestrial ancestors (Fig. 1A) through successive geological intervals. Finally, we fit a suite of phylogenetic comparative models to the temperature and precipitation optima inferred for every internal node state and project these evolutionary niches onto paleogeographic reconstructions of the last 245 Myr.

With these data in hand, we address four overarching questions: (i) which components of climate (temperature versus precipitation) enforced the strongest phylogenetic signal in Pseudosuchia, and how deep in the tree did that signal appear? (ii) What were the macroevolutionary effects of major Earth System upheavals, comprising the end-Triassic and end-Cretaceous mass extinctions, Mesozoic-Cenozoic hyperthermals, and mid-Cenozoic cooling, on pseudosuchian climatic niche optima and geographic range? (iii) Do climatic trait distributions across Pseudosuchia reflect a Brownian process of unconstrained macroevolutionary diffusion, or an Ornstein-Uhlenbeck (OU) process of bounded evolution, consistent with intrinsic constraints limiting the total trait space accessible to the clade, with episodic shifts in the long-term optimum tied to major geological events? (iv) To what extent do ancestral climatic reconstructions derived from phylogenetic comparative methods and ENM projections corroborate each other, and where do they diverge in ways that are informative about past niche limits or sampling gaps?

Together, this combined paleophylogeographic and ecological modeling approach lets us track how climatic preferences have been inherited, shifted in adaptive space, and/or lost in crocodylians and their ancestors over nearly a quarter-billion years and how their climatic niches were reflected in their paleobiogeographic history. By integrating fossil, phylogenetic, and climatic evidence at an unusually broad spatiotemporal scale, we provide the most quantitative test to date of climatic niche conservatism across the full evolutionary history of Pseudosuchia, characterize the timing and nature of regime shifts in both temperature and precipitation optima, and identify the geological events most consistently associated with transitions in macroevolutionary niche dynamics.

## RESULTS

### Macroevolutionary model fitting for Pseudosuchia

Both climatic axes show significant phylogenetic signal, with the high-λ/low-*K* combination diagnostic of multi-optima OU evolution (*10*) (Fig. 2, A to C). For mean annual temperature (MAT), Pagel’s λ = 0.99 (*P* < 0.001) and Blomberg’s *K* = 0.334 (*P* < 0.01). For mean annual precipitation (MAP), λ = 0.91 (*P* < 0.001) and *K* = 0.25 (*P* < 0.01). Both λ values approach the Brownian motion (BM) expectation of 1, indicating strong phylogenetic structure, while both *K* values fall well below 1, indicating that within-clade variance (Fig. 3, A and C) is compressed below the BM prediction, the pattern expected under multi-optima OU (Fig. 3, B and D). Trait evolution is characterized by bounded attraction toward long-term optima, with discrete clade-level regime shifts at specific geological boundaries (Figs. 2, D and E, and 3). For MAT, a primary shift is detected at the Cenomanian/Turonian (C/T) transition (∼93.9 Ma; Fig. 3, A and B, cumulative PP = 0.61), occurring within the early Crocodylia crown: Bayou locates the shift on the Alligatoroidea stem (∼90.3 Ma, θ = 35°C, PP = 0.62; Fig. 3, A and B), whereas SURFACE recovers it within Crocodyloidea (∼94.9 Ma; Fig. 3A). For MAP, a primary regime shift is detected at the E/O transition (∼33.9 Ma; Fig. 3E, cumulative PP = 0.59), with bayou locating a clade-level shift in the Australasian mekosuchine crocodyloid lineage (θ = 225 mm, PP = 0.58; Fig. 3, C and D), whereas SURFACE recovers only a terminal shift at this boundary, indicating that the precipitation niche reorganization is more restricted in phylogenetic extent than the widely distributed C/T thermal signal, and consistent with endemic Australasian crocodylians tracking the progressive Cenozoic aridification of that continent.

**Fig. 2.**
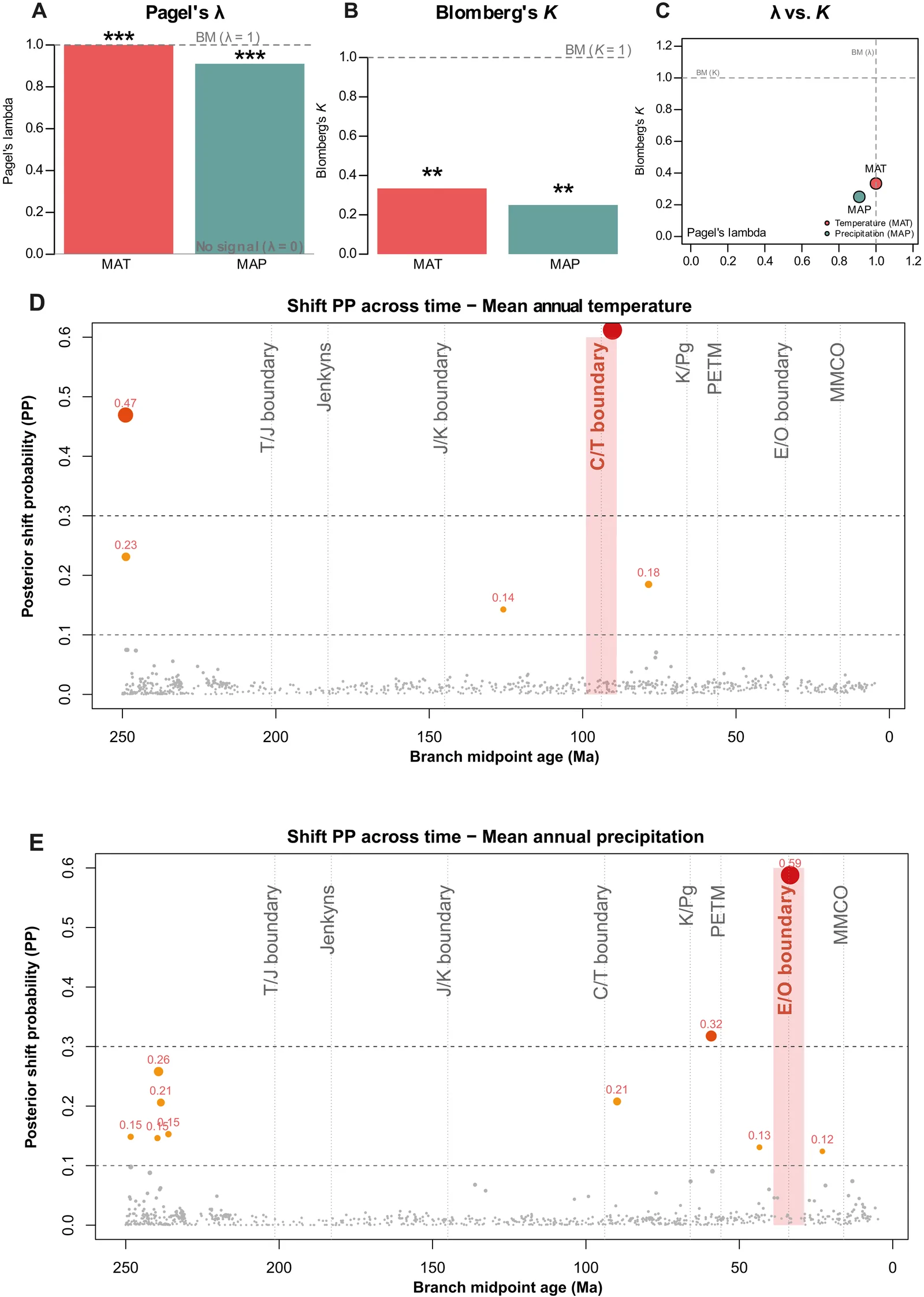
PNC in Pseudosuchia. (**A** and **B**) Parameters used to estimate PNC, Pagel’s λ, and Blomberg’s *K* for mean annual temperature (MAT) and mean annual precipitation (MAP), showing strong phylogenetic structure but trait disparity below Brownian expectations (dashed line). Asterisks indicate significant departures from λ = 0 or *K* = 0. (**C**) Combined λ–*K* results place both MAT and MAP in the high-λ/low-K region, consistent with bounded, multi-optimum niche evolution. (**D** and **E**) Posterior shift probabilities through time for MAT and MAP. The strongest MAT shift is concentrated near the Cenomanian/Turonian boundary (∼93.9 Ma), whereas the strongest MAP shift occurs near the Eocene/Oligocene boundary (∼33.9 Ma), indicating temporal decoupling of thermal and precipitation niche evolution in Pseudosuchia.

**Fig. 3.**
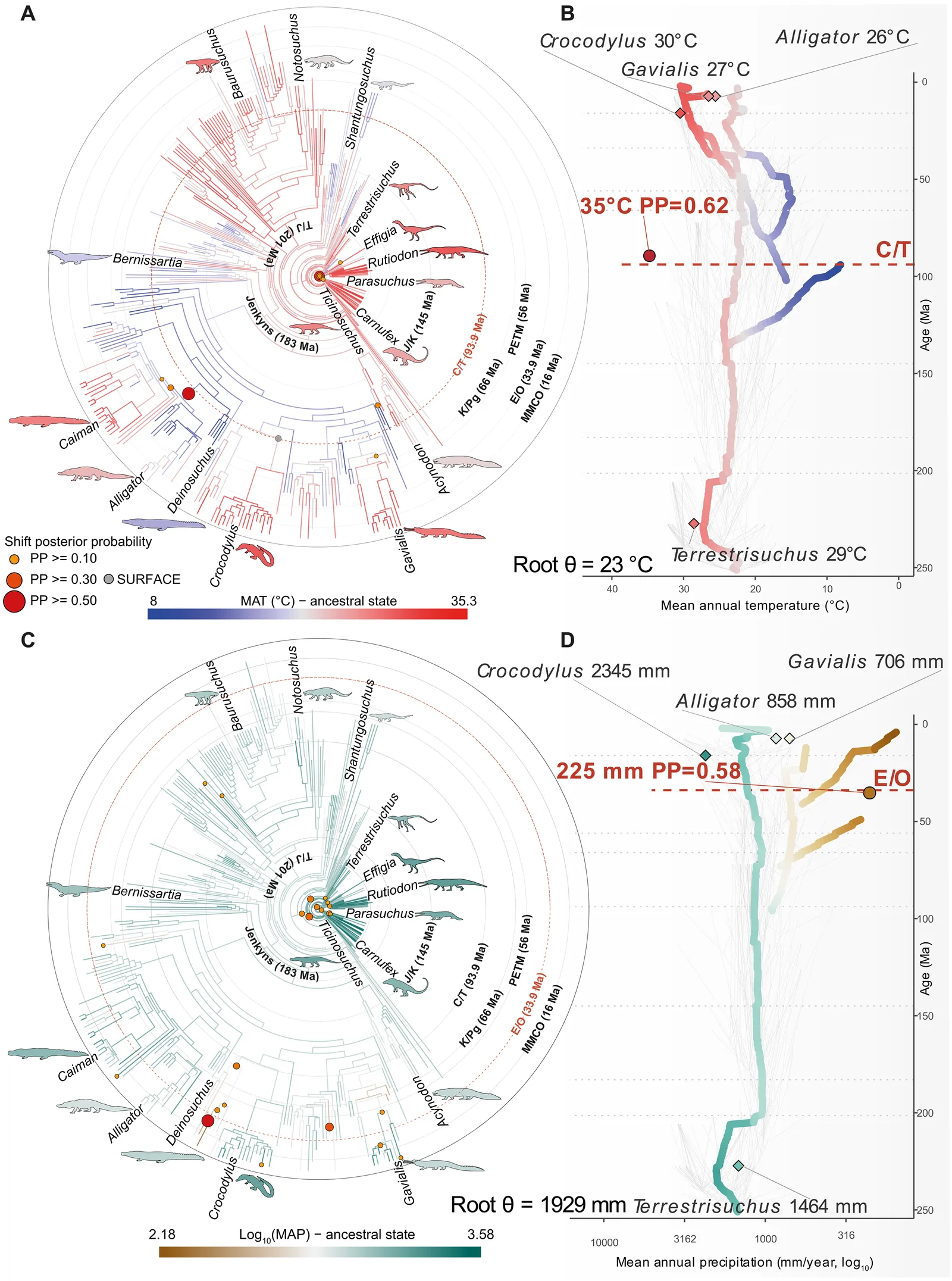
Climatic niche evolution in Pseudosuchia. (**A**) Circular time-calibrated phylogeny of Pseudosuchia with branches colored by reconstructed ancestral MAT under the best-supported single-regime OU model. Colored branch circles indicate bayou regime-shift posterior probabilities, and the gray circle marks the SURFACE-inferred internal-node shift. Selected tip taxa and geological reference boundaries are shown. (**B**) MAT phenogram showing reconstructed temperature trajectories through time, with the strongest regime shift concentrated near the Cenomanian/Turonian boundary (∼93.9 Ma). (**C**) Equivalent circular phylogeny for MAP, with branches colored by reconstructed ancestral precipitation values and the primary bayou shift located near the Australian mekosuchine lineage. (**D**) MAP phenogram showing reconstructed precipitation trajectories through time, with the strongest regime shift concentrated near the Eocene/Oligocene boundary (∼33.9 Ma). T/J, Triassic/Jurassic; Jenkyns, Jenkyns Oceanic Anoxic Event; J/K, Jurassic/Cretaceous; C/T, Cenomanian/Turonian; K/Pg, Cretaceous/Paleogene; PETM, Paleogene-Eocene Thermal Maximum; E/O, Eocene/Oligocene; MMCO, Middle Miocene Climatic Optimum. Organism silhouettes are from PhyloPic (https://www.phylopic.org/; T. Michael Keesey, 2023) and were added using the rphylopic R package version 1.7.0 (*219*). Organism silhouettes are from PhyloPic (https://www.phylopic.org/; T. Michael Keesey, 2023) and were added using the rphylopic R package version 1.7.0 (*219*). Silhouettes were made by Dan Folkes, 2025 (CC BY 4.0), Ananth Srinivas, 2025 (CC0 1.0), Scott Reid, 2021 (CC BY 3.0), Slate Weasel, 2011 (Public Domain Mark 1.0), Smokeybjb, 2011 (CC BY 4.0), Scott Hartman, 2011 (CC BY 3.0), Chase Brownstein, 2016 (CC BY 3.0), François-Louis Pelissier, 2023 (CC0 1.0), Jose Carlos Arenas-Monroy, 2022 (CC0 1.0), An Ignorant Atheist, 2019 (CC0 1.0), Edwin Price, 2023 (CC BY 4.0), and David Stanley, 2024 (CC BY 4.0). Silhouettes were contributed by Scott Reid, T. Michael Keesey, chasethedinosaur, François-Louis Pelissier, Dan Folkes, Jose Carlos Arenas-Monroy, Edwin Price, Scott Hartman, Ananth Srinivas, Jody Taylor, and Pascal Abel.

Crown-group temperature and precipitation estimates under OU show markedly lower dispersion than under BM or Early Burst (EB) (*30*), consistent with the bounded stationary variance of the fitted OU process. The OU optimum (θ) for the root of Pseudosuchia is estimated at 23°C (MAT) and 1929 mm (MAP), with crown-group tip optima spanning 26 to 30°C for MAT and 706 to 2345 mm for MAP across the three major extant lineages (Fig. 3, B and D). The fitted single-regime OU parameters for MAT are as follows: α = 0.0174 [95% confidence interval (CI): 0.0123 to 0.0235], θ = 23.1°C, σ^2^ = 1.320 (95% CI: 1.077 to 1.660); the phylogenetic half-life (ln 2/α) is 39.9 Ma (95% CI: 29.5 to 56.4 Ma), giving a ratio to clade age (252 Ma) of 0.16, indicating weak global attraction: MAT evolution approaches a near-Brownian regime at the total-group scale, with discrete shifts in θ across subclades. For MAP (log_10_-transformed), α = 0.078, θ = 1929 mm, σ^2^ = 0.0056; half-life = 8.9 Ma (95% CI: 4.2 to 14.8 Ma), ratio = 0.04. The MAP half-life is more than four times smaller than that of MAT, confirming that precipitation niche is under substantially tighter constraint than temperature niche throughout pseudosuchian history: Any lineage returns to >97% of its precipitation optimum within approximately 27 Myr. The stationary variance [σ^2^/[2α] = 1.320/0.035 ≈ 37.9°C^2^ for MAT; 0.0056/0.156 ≈ 0.036 log_10_(mm)^2^ for MAP] is markedly lower than the unbounded variance BM would produce at equivalent timescales, whereas BM and EB permit progressively broader dispersion among lineages. Together, these results demonstrate that climatic trait variance across Pseudosuchia is notably more bounded than unconstrained diffusion predicts, consistent with an OU process operating throughout the 250-Myr history of the clade, punctuated by discrete shifts in thermal and precipitation optima at or near the C/T and E/O boundaries, respectively (Fig. 3). Because OU is the best-supported process for both variables, OU-informed ancestral state reconstructions were used to derive climatic envelopes for paleogeographic projections (*31*). OU-based ancestral reconstructions were thus used for all paleogeographic projections of habitat suitability [phylogenetic paleophylogeographic modeling (PPGM) (*31*) approach; see Materials and Methods; Fig. 4], ensuring that mapped suitability reflects the bounded stationary variance of the best-fitting evolutionary model.

**Fig. 4.**
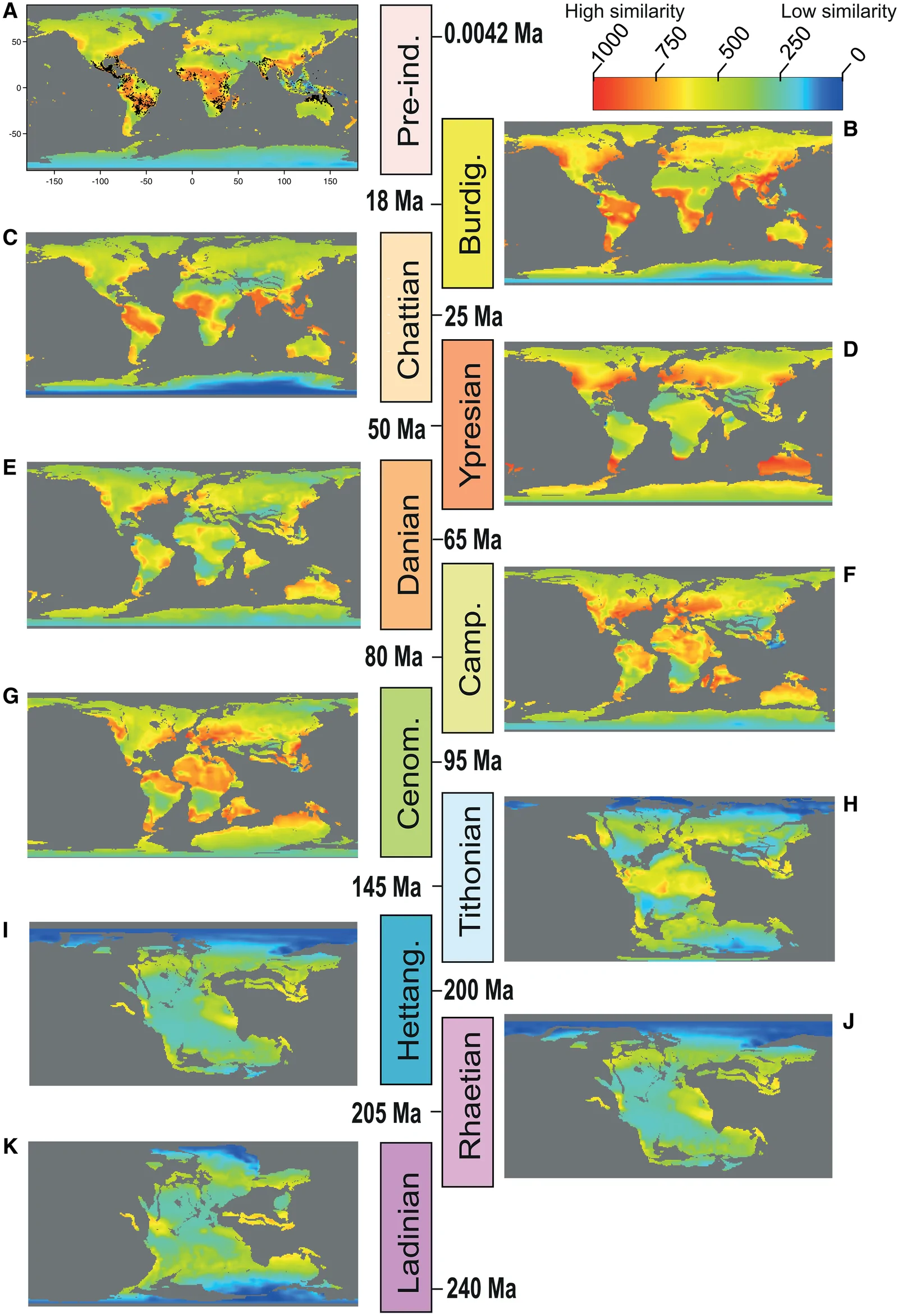
Phylopaleogeographic projections of pseudosuchian abiotic niche. PPGM suitability maps, indicated as similarity with extant available niche space, projected onto time-specific paleoclimate reconstructions. Suitability values are based on ancestral climatic envelopes reconstructed under the best-supported OU model and range from low to high suitability. Panels are arranged from youngest to oldest: (**A**) Pre-industrial, (**B**) Burdigalian, (**C**) Chattian, (**D**) Ypresian, (**E**) Danian, (**F**) Campanian, (**G**) Cenomanian, (**H**) Tithonian, (**I**) Hettangian, (**J**) Rhaetian, and (**K**) Ladinian.

### Spatiotemporal habitat suitability and the fossil record of Pseudosuchia

We reconstructed the fundamental climatic niche of total-group Pseudosuchia by coupling phylogenetic paleoclimatic reconstructions (PPGM) with ENMs trained on living crocodylians, projecting both across ∼245 Myr and global paleogeographies (Figs. 4 and 5). ENM-PPGM agreement in projections is high across most of the Mesozoic and early Cenozoic: Both methods recover poleward range expansions during warm phases, equatorward contractions during cool phases, and long-term confinement to low-mid paleolatitudes punctuated by poleward dispersal “windows.” Where they diverge, the nature of disagreement varies by time interval. In most Cretaceous and Paleogene slices, PPGM extends suitability into higher-latitude margins than ENMs, reflecting the capacity of PPGM to reconstruct fundamental niches beyond the modern realized niche that bounds ENM extrapolations. In some time slices, such as the Chattian (late Oligocene), the divergence appears more pronounced. This likely reflects stricter overfitting via PPGM between the maximum and minimum (*31*) values experienced by pseudosuchian lineages during that interval, compared with the broader fundamental niche modeled by ENM. ENMs project broader areas of high suitability in temperate regions, including western North America, southern South America, and southeastern Australia, which PPGMs instead recover as low-suitability areas. Conversely, PPGMs predict high suitability in tropical regions where ENMs are more conservative.

**Fig. 5.**
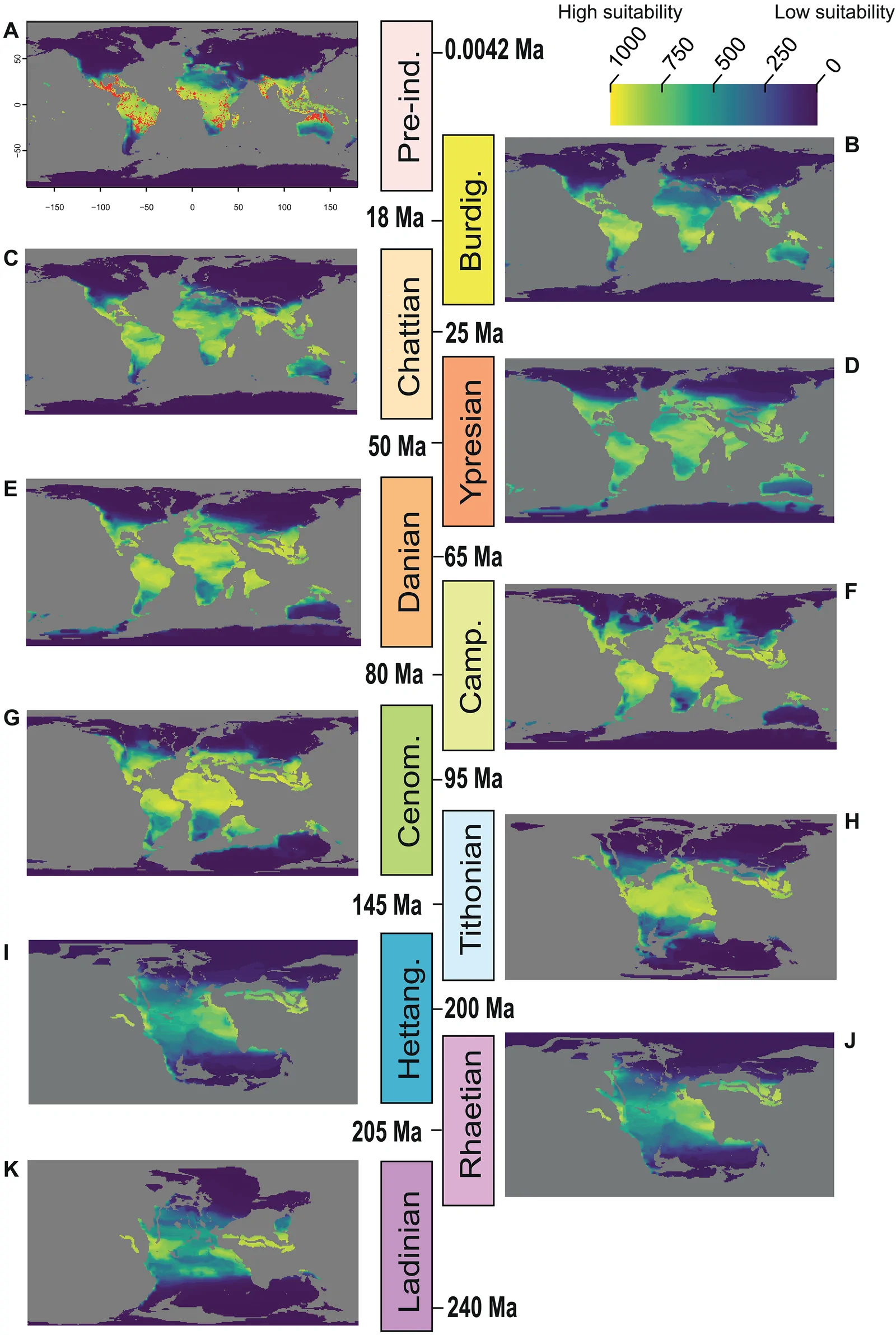
Paleogeographic projections of pseudosuchian abiotic niche from ENMs. TSS-weighted ensemble mean ENM suitability projected onto time-specific paleoclimate reconstructions. Models were calibrated using present-day occurrences of all extant crocodylian species and projected across four bioclimatic predictors for each time slice. Suitability values range from low to high, and red points in (A) indicate the present-day occurrence records used for model calibration. Panels are arranged from youngest to oldest: (**A**) Pre-industrial, (**B**) Burdigalian, (**C**) Chattian, (**D**) Ypresian, (**E**) Danian, (**F**) Campanian, (**G**) Cenomanian, (**H**) Tithonian, (**I**) Hettangian, (**J**) Rhaetian, and (**K**) Ladinian.

Both frameworks recover extensive suitability in the Early Triassic, the hypothesized origin time of stem Pseudosuchia, centered at low-mid paleolatitudes (Figs. 4K and 5K). We note that the Ladinian time slice represents the deepest time point in our panel sequence and consequently the greatest departure from modern climate analog conditions (Fig. 4K); PPGM predictions are therefore weighted more heavily for this interval. This is consistent with ichnological evidence that extends the pseudosuchian record back into the Early-Middle Triassic (∼252 to 237 Ma), indicating widespread presence along low-latitude corridors well before the Carnian-Norian body-fossil peak (*32*–*39*), and aligns with proposals for a low-latitude origin (*39*). Suitability rises through the Carnian (237 to 227 Ma), expanding from the paleoequator and likely reflecting humidity increases tied to the Carnian Pluvial Event (∼234 to 232 Ma) (*40*–*44*). Suitability peaks in the Norian (227 to 208.5 Ma) and contracts toward the Triassic/Jurassic (T/J) boundary (∼201.3 Ma; Fig. 2C). Late Triassic body fossils support a low-to-mid-latitude suitability belt across Laurasia and southern Gondwana (*45*–*53*), with gaps in Arabia–northeastern Africa consistent with undersampled dispersal corridors within which we predict the presence of recoverable Late Triassic pseudosuchians.

Modeled suitability is low across the T/J boundary, with a Rhaetian-Hettangian (205 to 199.3 Ma) minimum in equatorial inlands and with greater suitability on the western coastal belts. Suitability then recovers in the Sinemurian (199.3 to 190.8 Ma) and especially the Pliensbachian-Toarcian (190.8 to 174.1 Ma). The fossil record broadly tracks this pattern: Hettangian–early Sinemurian localities are scarce, whereas Pliensbachian-Toarcian assemblages are rich. Apparent lags are most plausibly preservational: Targeted work in Hettangian equatorial coastal-fluvial refugia and earliest Sinemurian basins along inferred corridors could test whether high suitability in these areas corresponds to high but currently unsampled crocodyliform paleodiversity. Suitability rises through the Middle Jurassic, with an interior-Gondwana maximum during the Bathonian–early Oxfordian (168.3 to 161 Ma), and remains extensive and comparatively stable through much of the Late Jurassic (Fig. 4H). PPGM consistently allows broader high-latitude windows (northern Laurasia; parts of Gondwana, including northern Australia, Antarctica, India) than ENM, but both agree on persistent paleoequatorial-tropical suitability. Sustained mid- to high-latitude suitability in the Northern Hemisphere is supported by fossil occurrences in both western and eastern Laurasia (*54*–*61*). Model outputs imply a near-continuous eastern-Gondwanan corridor across central-eastern Africa linking southern Africa (*62*, *63*) and Asia at the same time; however, most of this Gondwanan region is poorly sampled for Middle-Late Jurassic terrestrial faunas. Australia and Antarctica lack confirmed Jurassic terrestrial crocodylomorph body fossils (*64*). Given the modeled windows, small-bodied terrestrial crocodyliforms are plausible targets in Indian Gondwana and Antarctic basins, where taphonomic conditions are favorable (*65*). With suitability broadly stable across the Jurassic/Cretaceous boundary, the Berriasian-Valanginian (145 to 132.9 Ma) deposits of western Europe provide a rich non-marine crocodyliform assemblage (*66*, *67*). Contemporaneous deposits in China are dominated by ichnofossils (*68*, *69*), suggesting that body fossils should be recoverable in equivalent beds of northern China/southern Mongolia (*70*). Limited sampling opportunity might explain their scarcity elsewhere, although it remains possible that they were genuinely rare in the earliest Cretaceous (*57*, *71*). From the early Aptian (∼120 Ma) to mid-Albian (∼110 Ma), suitability contracts latitudinally (losses from continental interiors and latitudinal extremes) and then recovers in the late Aptian and into the Cenomanian (100.5 to 93.9 Ma). The Cenomanian terrestrial record remains sparse despite modeled high suitability (Figs. 4G and 5G, plausibly due to the C/T sea-level highstand, 93.9 to 89.8 Ma, suppressing inland floodplain preservation (*72*–*74*). Where present, Cenomanian continental strata cluster in western US microsites (*75*), along Tethyan/northcentral Gondwanan margins (*76*–*78*), and the southeastern Gondwanan margin (*79*), supporting an equatorial-subtropical corridor from Africa to eastern Gondwana (*64*, *80*–*82*). Turonian-Santonian suitability is broadly stable but drifts slightly equatorward in the Campanian-Maastrichtian (83.6 to 66.0 Ma; Figs. 4F and 5F), with a moderate decline in suitable area and reduced Northern Hemisphere spread. Fossils indicate a genuinely broad warm-belt fauna, not confined to North America: Crocodyliforms are widespread in Europe, Asia, North (up to ∼67°N) and South America, and Madagascar (*83*–*94*). This circumtropical-subtropical to warm-temperate distribution matches ENM-PPGM suitability ranges and latitudes. Additional Maastrichtian material is anticipated in paleoequatorial Africa/Asia, including Indian intertrappeans (*27*, *95*, *96*).

To assess pseudosuchian habitat suitability through the K/Pg boundary, we performed a suite of coupled atmosphere-ocean general circulation model (AOGCM) transient forcing experiments (Fig. 6), following the approach of Chiarenza *et al.* (*27*). These experiments are designed to capture the short-duration, decadal-to-centennial climatic perturbations associated with the Chicxulub asteroid impact, a fundamentally different timescale from the ∼5-Myr resolution of the long-term ENM and PPGM projections, which are not designed to resolve transient events. Each simulation was branched from a fully equilibrated Maastrichtian climate baseline and subjected to one or more of the following forcings: (i) asteroid impact-induced solar dimming at three magnitudes (−5%, −10%, and −15% reduction in solar constant; Fig. 6, C to E), corresponding to a range of ejecta opacity estimates; (ii) particulate ash-layer deposition (×50 and ×100 present; Fig. 6, F and G); (iii) Deccan Traps CO_2_ enrichment at two levels (2× → 4× and 2× → 6× pre-industrial CO_2_; Fig. 6, I and J); and (iv) a combined impact-plus-Deccan scenario (Fig. 6K). ENM suitability was then projected onto each resulting climate field to evaluate which forcing scenarios, if any, eliminated climatically suitable habitat for pseudosuchians. These simulations indicate persistence of low-latitude climatically suitable refugia across all but the most extreme impact scenario: Only the −15% solar-dimming case (Fig. 6E) eliminates suitable climates globally. Deccan-driven CO_2_ enrichment (Fig. 6, I and J) expands latitudinal suitability, and combined transient forcings (Fig. 6K) do not eradicate the pseudosuchian climatic niche.

**Fig. 6.**
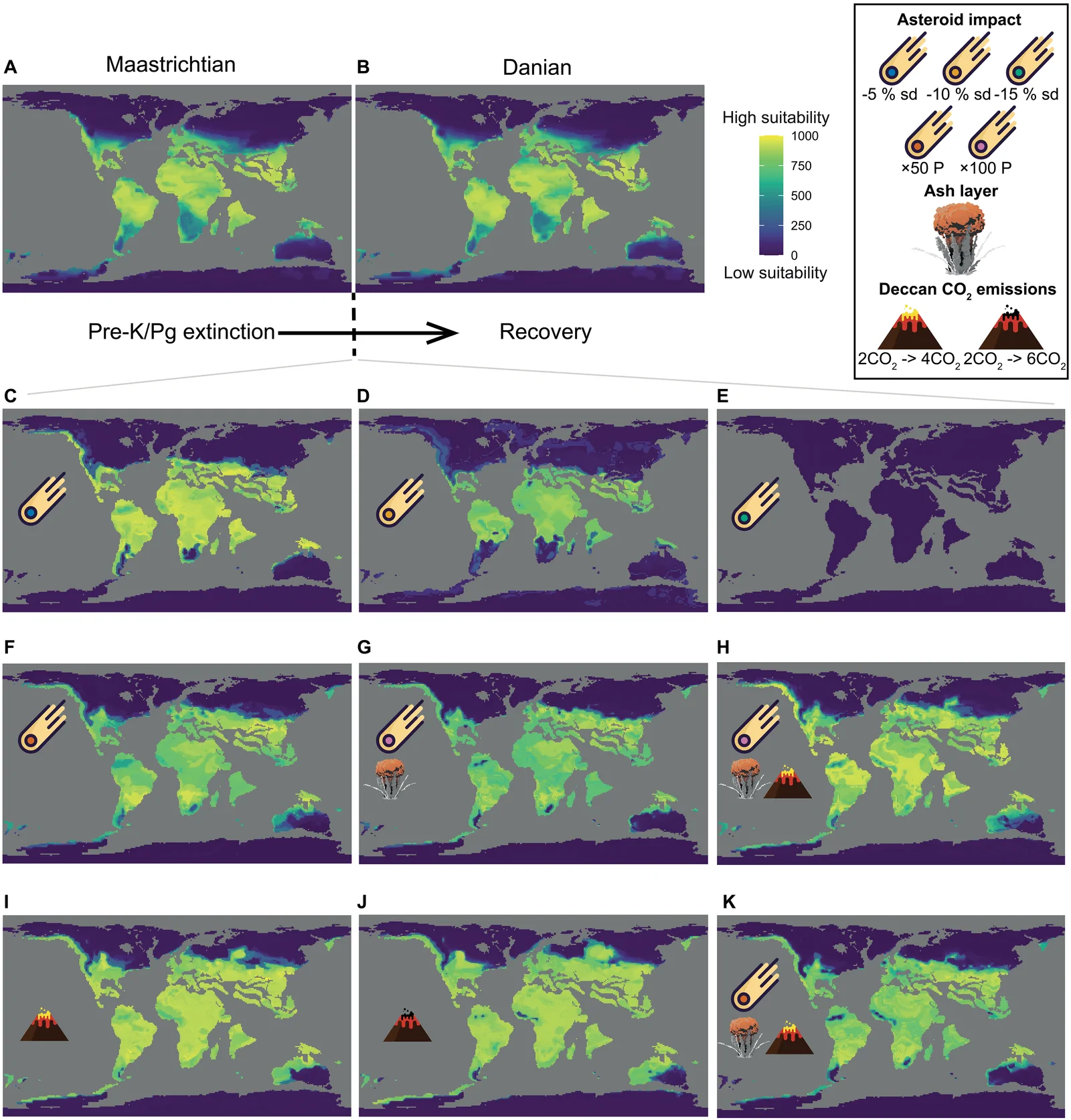
K/Pg experiments on pseudosuchian climatic suitability. (**A**) Ensemble ENM projections on Maastrichtian and (**B**) Danian paleogeography. Asteroid impact induced solar-dimming (sd) scenarios: (**C**) −5%, (**D**) −10%, and (**E**) −15% relative to the Maastrichtian solar constant. Transient impact aerosol scenarios with Pinatubo (P) scaled forcing with ash layer over North America: (**F**) 50× P + ash layer, no Deccan; (**G**) 100× P + ash layer; (**H**) 100× P + ash layer + concurrent Deccan CO_2_ rise 2× → 4×. Longer-term CO_2_ increases from Deccan volcanism with no solar change (**I**) 2× → 4× and (**J**) 2× → 6×. (**K**) Combined impact + Deccan: 50× P + ash + 2× → 4×.

Danian (66.0 to 61.6 Ma) suitability (Figs. 4E and 5E) rebounds to near-Maastrichtian levels and stabilizes through the Selandian-Thanetian (61.6 to 56.0 Ma). Danian occurrences in the Gulf region, northwestern Africa, and northern South America align with recovery within tropical refugia (*95*–*100*). A marked Ypresian (56.0 to 47.8 Ma) expansion pushes suitability poleward (Figs. 4D and 5D), coincident with the PETM (∼56 Ma) and the subsequent Early Eocene Climatic Optimum (EECO; ∼53 to 49 Ma), and is mirrored by high-latitude records in North America, northwestern Europe, and Antarctica, with further gains in both hemispheres (*101*–*107*). The Ypresian (Figs. 2D and 3D) captures the apex of this expansion and documents the broadest poleward extent of pseudosuchian habitat suitability in the Cenozoic.

Suitability remains elevated through the Lutetian-Bartonian (47.8 to 37.8 Ma) and declines slightly by the Priabonian (37.8 to 33.9 Ma). A pronounced contraction follows, with minima in the Chattian (27.8 to 23.0 Ma; Figs. 4C and 5C). Persistent low-latitude refugia (western Atlantic/Gulf margins; western-north Africa; Afro-Arabia to the northern Indian Ocean rim) and small paleotemperate pockets around the Paratethys are recovered; high-latitude windows contract strongly in both hemispheres (Figs. 2C and 3, C and D). Fossils from Europe, northern Africa, North America, and southeastern Asia (*108*–*113*) mirror these trends. South America shows a sampling trough despite comparatively high, stable suitability; Antarctica lacks Oligocene crocodyliform body fossils; Australia retains moderate but slightly declining suitability, consistent with its fossil record (*114*, *115*). Miocene models indicate renewed expansion tied to the Mid-Miocene Climatic Optimum (Burdigalian–Langhian/Serravallian, 20.4 to 13.8 Ma), supported by a globally well-sampled fossil record (Figs. 1F, 4B, and 5B) (*111*, *116*–*126*). Zanclean models (5.33 to 3.6 Ma) maintain high suitability in the southern United States (*127*), southwestern South America (*128*), south-central Africa (*129*), India (*130*), and Australia, and flag additional suitable climates in potentially undersampled regions (e.g., northwestern USA; some European coastal localities). PPGM yields broader high-latitude windows than ENM, including Japan, New Zealand, and southernmost South America.

Pre-industrial projections reproduce the modern tropical-equatorial distribution in both frameworks (Figs. 4A and 5A), with tighter fidelity on modern occurrences in ENM models, whereas PPGM highlights some overpredicted areas at higher temperate localities, such as in South America and New Zealand, or currently unfilled windows in central-western Europe. Because climate surfaces utilized herein operate at ∼5-Myr resolution, short-period range dynamics (e.g., Quaternary oscillations) cannot be resolved. Pleistocene–early Holocene records extend to higher latitudes than today in several regions, consistent with ephemeral extra-tropical incursions (*131*–*135*), although the absence of crocodylians from continental Europe from the Pliocene onward most plausibly reflects genuine regional extirpation during cooler phases of the last ∼5 Myr.

## DISCUSSION

### A bounded climatic niche through deep time

Our results indicate that the climatic niche of Pseudosuchia evolved in a manner best captured by an OU process (Fig. 3). In a uniform OU model, both the rate of attraction toward the optimum and the stochastic variance are time-invariant parameters that apply equally throughout the phylogeny (*136*). The signature of the fitted model is not that dynamics change through time, but rather that the stationary variance is demonstrably lower than the unbounded variance that BM would produce across 250 Myr of independent evolution. This means that trait disparity is consistently bounded relative to the BM expectation at any timescale we examine throughout the 250-Myr history of Pseudosuchia.

These results are consistent with PNC [sensu Harvey and Pagel (*9*)] across Pseudosuchia. The phylogenetic half-life indicates bounded climatic niche evolution over geological timescales, with precipitation showing especially strong resistance to sustained directional shifts. Rather than tracking each climatic perturbation through wholesale niche displacement, pseudosuchian lineages appear to have retained broadly similar climatic optima across intervals comparable to, or longer than, many major Mesozoic and Cenozoic environmental events. The warm thermal optimum inferred for the clade, together with the restricted tropical-subtropical climatic range occupied by living crocodylians, suggests that the modern crocodylian niche is not merely a recent ecological specialization, but the surviving expression of a much deeper pseudosuchian climatic signature (*3*, *10*, *11*, *137*). The fitted thermal optimum for the pseudosuchian root (θ = 23.1°C) and the crown-group tip range (26 to 30°C for the three extant lineages; Fig. 3B) are both concentrated in warm tropical-subtropical conditions, narrower than the global temperature range occupied by birds (broadly −20 to 30°C) (*13*) or mammals (*137*), consistent with PNC in both climatic scale and temporal extent.

Within this bounded macroevolutionary regime, our multi-regime analyses identify two geological boundaries at which discrete shifts in the long-term optimum (θ) occurred, one for each climatic axis and at separate moments in geological history. For MAT, the primary clade-level shift is associated with the C/T boundary (Figs. 2D and 3, A and B), independently identified by both bayou and SURFACE. The C/T boundary coincides with Oceanic Anoxic Event 2 (OAE2), one of the most severe hyperthermal events of the Phanerozoic, with sea-surface temperatures exceeding 35°C regionally (*138*). The shift in θ at this boundary, from the root optimum of ∼23°C to ∼35°C, reflects a reorganization of the thermal optimum within the early crown-crocodylian radiation during one of the most extreme warming events in the Mesozoic, rather than a global niche shift across all Pseudosuchia. Following this interpretation, the C/T Thermal Maximum would have created strongly differentiated selective regimes across latitude, with lineages centered at contrasting thermal positions experiencing very different fitness landscapes simultaneously.

In contrast to the broader C/T thermal signal, the MAP shift near the E/O boundary appears phylogeographically restricted to the Australasian mekosuchine crocodyloid lineage, indicating a more regional response rather than a reorganization of the crown radiation as a whole. This narrower signal is nevertheless consistent with the broader climatic context of the E/O transition, the largest global aridification event of the Cenozoic, which drove major contraction of tropical forest belts and expansion of open, dry biomes (*139*, *140*); the shift toward drier precipitation optima in Australasian mekosuchines at this boundary is therefore consistent with these endemic crocodylians tracking the progressive aridification of the Australian continent (*141*), which has been one of the most dramatically affected landmasses by Cenozoic drying (*142*).

The two regime shifts are temporally decoupled: The C/T thermal signal is absent from MAP models (bayou PP ≈0 at CT for MAP) and the E/O precipitation signal is absent from MAT models (PP ≈0 at EO for MAT). Temperature and precipitation niches were therefore reorganized at separate geological moments, driven by fundamentally different Earth System events: a “mid-Cretaceous” thermal maximum for temperature and a Cenozoic aridification transition for precipitation. Neither axis is a passive correlate of the other. Prior work documenting pseudosuchian thermophily (*20*, *21*) necessarily focused on temperature because the primary signal in extant distributions is thermal, but the MAP analysis reveals an independent and geologically more recent constraint that has not previously been characterized quantitatively.

The restricted and geographically patchy suitability recovered across the latest Triassic–earliest Jurassic provides a climatic context for the selective collapse of pseudosuchian diversity at the T/J boundary. Multiple diverse lineages, including phytosaurs, aetosaurs, and rauisuchians, disappeared across this interval (*143*–*145*), with only small-bodied members of the crocodyliform lineage persisting and subsequently diversifying in the Jurassic (*146*–*149*). The reduction in both taxonomic and geographic sampling of pseudosuchian climatic space is consistent with the limited suitability recovered through the Hettangian. Subsequent increases in suitability track the Jurassic diversification of crocodyliforms into new ecomorphologies and regions (*146*–*149*), apparently driven by climatic amelioration rather than a shift in the underlying climatic optimum, as no OU regime shift is detected at or near the T/J in either bayou or SURFACE.

From the Jurassic onward, ENM and PPGM projections converge on repeated openings of dispersal and suitability “windows” at higher paleolatitudes during warm phases (e.g., Middle-Late Jurassic, early Late Cretaceous, early Eocene, and middle Miocene), expressed as poleward range extensions of suitable habitat and pseudosuchian distribution, rather than sustained shifts in thermal optima, i.e., extrinsic (paleoclimatic) forcing acting on an intrinsically conservative niche. Consistent with this interpretation, ranges contract equatorward, while reconstructed optima remain within a narrow latitudinal band during cooling intervals (e.g., the E/O and Pliocene-Holocene transitions). The exceptions to this pattern of pure range tracking, i.e., the C/T thermal shift at the base of Crocodylia (considering both the bayou Crocodyloidea and the SURFACE Alligatoroidea shifts; Fig. 1) and the E/O precipitation shift in Australasian mekosuchines, are the only intervals at which the underlying optimum itself, rather than merely its geographic expression, appears to have changed, and both are detected independently by two analytical frameworks operating without any prespecified boundaries. This pattern of geographic range expansion and contraction, while the range of occupied climatic trait values are significantly more bounded than unconstrained diffusion would predict, is consistent with intrinsic physiological limits and/or macroevolutionary constraints limiting the total niche space accessible to the clade, with dispersal facilitating geographic tracking of suitable climates as they shift. We note that “bounded” here is relative to the broader diffusion of BM expectations: The occupied temperature range across all OU regimes (∼15 to 41°C; Fig. 3B) is broader than that of individual living crocodylian species, but narrower than BM would predict over 250 Myr, an expectation of PNC (*10*, *150*).

We cautiously remark that PNC describes a pattern, not a single process (*151*), and multiple other mechanisms, including local extinctions, genetic/developmental constraints, and trade-offs among co-adapted traits, can further affect and produce, in concert, evidence of niche conservatism at micro-(species level) to macroevolutionary (supra-specific clade) scales (*152*–*154*). Because ENMs are calibrated on realized niche space, they typically yield more conservative multivariate niches than PPGM projections of the broader fundamental niche (*155*, *156*).

OU-constrained PPGM reconstructions produce ancestral climatic envelopes bounded by the stationary variance throughout the pseudosuchian phylogeny, a property of the model applying from the root to tips represented by crown Crocodylia. This explains the close agreement between ENM projections for present-day crocodylians and PPGM-derived suitability across most of pseudosuchian history (Figs. 4 and 5): Because pseudosuchian climatic niches are bounded by the OU stationary variance, ancestral niches reconstructed by PPGM do not project far into high-latitude cool climates, where fossils are absent (*21*). A BM-based PPGM would instead project expanding suitability into higher paleolatitudes at deeper timescales, contradicting the fossil record of persistent low-to-mid-latitude confinement.

### The role of a conservative climatic niche in pseudosuchian survival at the K/Pg boundary

Our paleoclimatic transient simulations (Fig. 6) consist of coupled climate model runs forced with Chicxulub-impact perturbations applied over decadal-to-centennial timescales to a fully equilibrated Maastrichtian baseline. Projecting the pseudosuchian climatic niche through these models shows that climatically suitable habitat for crocodile-line archosaurs persists across most extinction-forcing scenarios. The −15% solar-dimming scenario represents a sustained multi-year to decadal reduction in surface insolation at the extreme end of published Chicxulub ejecta opacity estimates (*27*, *157*), and is the only scenario that eliminates suitable climatic niche globally.

The K/Pg boundary simulations provide a decisive test of whether thermophilic niche conservatism functions as a liability or a buffer under an impact-winter scenario (*27*, *158*, *159*) (Fig. 6). High-resolution experiments show that suitable climates for crocodile-line archosaurs persist under plausible Chicxulub-impact forcings; only an extreme −15% solar-dimming eliminates them, a magnitude well beyond most estimates (*27*, *157*). Deccan CO_2_ enrichments broaden latitudinal suitability, and combined impact-volcanism transients do not extirpate suitable niches, with recovery emerging from within low-latitude refugia. These results support a spatial mechanism for pseudosuchian survivorship across the K/Pg: No abrupt adaptive shift was required because suitable climates likely persisted in equatorial and subtropical belts, even during short-lived impact-winter conditions (*157*). By contrast, abiotic niche space inferred for non-avian dinosaurs collapses globally in comparable experiments (*27*), consistent with the selective extinction of dinosaurian clades whose climatic envelopes lacked contiguous refugia. Under this scenario, the thermophilic OU optimum of pseudosuchians, centered at ∼23° to 35°C and geographically expressed in equatorial and subtropical belts, placed surviving lineages in exactly the regions where climatic suitability was most resilient to the K/Pg impact forcing.

We emphasize that habitat suitability modeling provides a necessary, but not sufficient explanation for K/Pg extinction selectivity. Non-abiotic factors—including smaller body size, semi-aquatic lifestyle providing access to detrital food webs less disrupted by productivity collapse (*27*, *28*), dietary generalism, and ectothermy reducing baseline metabolic demand—undoubtedly contributed to differential survival, and a pseudosuchian and a dinosaur sharing the same Maastrichtian environment presumably tolerated similar ambient temperatures. What our results highlight is that the spatial distribution of climatically suitable habitat under impact-winter forcing is consistent with the observed geographic pattern of pseudosuchian survival (*160*, *161*), and that no evolutionary shift in the climatic optimum was required for post-K/Pg recovery. In sum, our simulations demonstrate that the geographic distribution of climatically suitable habitat for pseudosuchians was resilient to all but the most extreme K/Pg-impact scenarios, providing a plausible abiotic mechanism for low-latitude refugial survival. Post-K/Pg recovery, a Danian rebound followed by early Eocene poleward expansion, coincident with the PETM and EECO hyperthermals (Figs. 4, D and E, and 5, D and E), arises from the geographic re-expression of the conserved thermal optimum as global temperatures ameliorated, not from any detectable shift in that optimum (Fig. 2, D and E).

### Using ENM in predicting long-lived clade dynamics through past and future climate changes

ENM-PPGM comparisons indicate a conserved, OU-bounded thermal regime operating throughout pseudosuchian history. Apparent discrepancies at the margins (notably, PPGM’s broader high-latitude windows in some intervals; Figs. 4 and 5) are informative rather than contradictory: They highlight (i) areas where fossil sampling is inadequate, (ii) occupied climates that approach the limits of the modern training space, and/or (iii) a methodological asymmetry between frameworks. Specifically, PPGM infers the clade’s fundamental climatic envelope from the phylogeny, taking into consideration minima and maxima of their occupied climatic envelope, and can project it into regions and climates (*31*) that are unsampled today. By contrast, ENMs are trained on realized modern ranges and therefore contract (or flag) predictions near or beyond the training domain. This mismatch results in broader edge predictions under PPGM and more conservative margins under ENM [e.g., relative to PPGM-predicted areas (*31*, *162*–*164*)], with some workers (*156*, *165*–*167*) considering ENM outputs closer (but not identical) to the realized niche.

Our results indicate that ENMs trained on the present-day distribution of crocodylians can reliably hindcast the climatic niche of their lineage through deep time because the niche itself is strongly conserved. Under the OU-constrained, reconstructed dynamic, the realized niche of living species is a reasonable proxy for the fundamental niche expressed by their fossil relatives. Consistent with this, maps of ENM suitability closely track PPGM projections and the sampled fossil record across major episodes: low-latitude Triassic suitability expanding through Jurassic recovery, Middle-Late Jurassic poleward windows, the early Eocene expansion into high latitudes, the Oligocene retreat, and renewed Miocene expansion. Even at the K/Pg boundary, ENMs forecast persistence in low-latitude belts and rapid post-impact recovery, patterns mirrored by early Paleogene fossil occurrences. An additional caveat concerns the deep-time Triassic time slices, where MESS values (Fig. 4) indicate the greatest departure from the modern climate-training domain and ENM extrapolation is therefore least reliable; PPGM predictions carry more weight in those intervals. Increased fossil sampling of Rhaetian-Hettangian terrestrial faunas could improve ancestral niche estimation accuracy and reduce PPGM-ENM divergence for that time interval.

Rather than undermining the approach, these mismatches provide targeted hypotheses for future exploratory work (*115*, *168*). Together, the concordance among ENMs, PPGMs, and the fossil record supports the temporal transferability of modern-trained ENMs for long-lived, physiologically conservative clades (*15*). The main caveats involve coarse temporal bins, non-analog climates, and preservational biases (*169*). Transferability may have more predictive power when projections are aimed at time slices after the last major shift in evolutionary regime experienced by the clade of interest.

More generally, the pseudosuchian example underscores that geographic range dynamics in this clade are mainly governed by the spatial availability of climatically suitable habitat operating on a conserved climatic niche, punctuated by two discrete shifts in the long-run optimum—one thermal at the C/T boundary and one related to precipitation at the E/O boundary—that reorganized the niche of specific subclades at geologically distinct intervals. The consilience between ENM, PPGM, and the fossil record across 250 Myr demonstrates that pseudosuchian climatic niche conservatism is a robust macroevolutionary signal, at least for the last 100 Myr, validated by multiple independent analytical frameworks, and provides a quantitative baseline for understanding how physiologically conservative clades respond to major climatic perturbations in the geological past.

## MATERIALS AND METHODS

We reconstructed the climatic niche of Pseudosuchia through time by combining (i) ENMs trained on occurrences and (paleo-)climate covariates and (ii) phylogenetic paleophylogeographic reconstructions [PPGM (*163*)] of climatic envelopes.

### Occurrence data assembly and filtering

We obtained present-day crocodylian occurrences from GBIF (GBIF.org) using the R package rgbif (*170*), querying the currently recognized extant species: *Alligator mississippiensis*, *Alligator sinensis*, *Caiman crocodilus*, *Caiman latirostris*, *Caiman yacare*, *Crocodylus acutus*, *Crocodylus halli*, *Crocodylus intermedius*, *Crocodylus johnstoni*, *Crocodylus mindorensis*, *Crocodylus moreletii*, *Crocodylus niloticus*, *Crocodylus novaeguineae*, *Crocodylus palustris*, *Crocodylus porosus*, *Crocodylus rhombifer*, *Crocodylus siamensis*, *Crocodylus suchus*, *Gavialis gangeticus*, *Mecistops cataphractus*, *Melanosuchus niger*, *Osteolaemus tetraspis*, *Paleosuchus palpebrosus*, *Paleosuchus trigonatus*, and *Tomistoma schlegelii*. We followed the protocol in Jones *et al.* (*171*) to assemble and vet fossil and present-day occurrence databases. We vetted records by basis-of-record to exclude fossils and preserved/museum specimens in present-day occurrences (and removed obvious non-native releases and zoo records). Following Chiarenza *et al.* (*16*, *27*, *43*, *172*, *173*), we reduced spatial autocorrelation by retaining a single occurrence per climate grid cell (18.5 × 18.5 km at the equator), yielding 1296 unique grid-cell presences for ENM calibration. We exported the vetted data as GBIF occurrence downloads (one per species), each with a citable DOI and an access date of 19 February 2025 (Table 1). Taxonomic names were validated against the GBIF Backbone Taxonomy (*174*), which we also cite with the same access date. All occurrences are available in the supplementary materials as (i) originally downloaded, per species occurrences, aggregated, unvetted occurrences, and (ii) vetted, spatially filtered occurrences. Comparisons between ecological niche projections in the past and fossil occurrences of pseudosuchians in these time intervals were based on data from the Paleobiology Database, available at this link: https://paleobiodb.org/data1.2/occs/list.csv?datainfo&rowcount&base_name=Pseudosuchia&interval=Ladinian,Holocene&pgm=gplates,scotese,seton&show=full,classext,genus,subgenus,acconly,ident,img,etbasis,strat,lith,env,timebins,timecompare,resgroup,ref,ent,entname,crmod. This database was checked, vetted, and edited for accuracy and congruence with the phylogeny of Pseudosuchia used in this study published by Payne *et al.* (*175*). Modern coordinates of localities where these fossil occurrence come from were rotated to paleo-coordinates based on the plate model used in this study (*176*, *177*) using the “palaeorotate” function in palaeoverse (*178*). The matured fossil occurrence dataset includes 6412 spatially unique-grid entries and is available, together with the raw data, at the following zenodo DOI 10.5281/zenodo.18354822.

### Paleoclimate data and paleogeographic framework

Temporal paleoclimatic projections span the Triassic to Pre-industrial. For deep-time intervals, we report stage-level results where appropriate but, following the resolution of the paleoclimate grids, we summarize maps and metrics at ∼5-Myr steps when stage reconstructions could not be uniquely resolved. We note that this temporal resolution is appropriate for characterizing long-term geological trends in habitat suitability, but is insufficient to resolve transient climatic perturbations such as impact-winter events or hyperthermal episodes lasting decades to millennia; those are addressed with dedicated short-duration simulations described below (see paleoclimate models of K/Pg scenarios). Climatic predictors were derived from coupled AOGCM simulations and matched paleogeographic reconstructions following deep-time ENM practice in Chiarenza *et al.* (*16*, *172*, *173*, *179*) and Foffa *et al.* (*43*). We used the HadCM3L-M2.1aD configuration with a 3.75° (longitude) × 2.5° (latitude) atmosphere and ocean grid, 19 hybrid atmospheric levels, and a three-dimensional primitive-equation ocean (20 vertical levels) solved on an Arakawa B-grid. Sub-grid atmospheric processes (e.g., moist convection) are parameterized; the ocean mode uses centered advection with a second-order numerical scheme (*180*). The model does not require flux adjustments, and sea ice is treated with a zero-layer thermodynamic scheme allowing partial grid-cell coverage. Land surface processes are represented by MOSES 2.1a coupled to the dynamic vegetation model, TRIFFID, which predicts fractional coverage of five plant functional types (C3 and C4 ground cover, shrub, broadleaf tree, needleleaf tree) from the simulated climate.

Geologic stage-specific boundary conditions included paleogeographic topography/bathymetry, land ice, and coastlines [PALEOMAP-based digital elevation models interpolated to the model grid (*176*)], and solar luminosity appropriate to each interval was calculated from Gough (*181*). Atmospheric carbon dioxide concentrations [partial pressure of CO_2_ (*P*co_2_)] for each time interval utilize realistic multi-proxy CO_2_ reconstructions derived from Rae *et al.* (*182*) between 69 and 0 Ma and from Foster *et al.* (*183*) between 245 and 69 Ma. Because globally consistent geologic soil data are unavailable for deep-time intervals, soils were set to a uniform medium loam. Because globally consistent geologic soil data are unavailable for deep-time intervals, soils were set to a uniform medium loam. Each equilibrium experiment was integrated for multi-millennial durations (>6000 model years), and climatological means were computed from the final 100 years of each time interval. Desert soil albedo is interactively updated on the basis of the soil carbon content, where low soil carbon concentrations result in a modified soil albedo of 0.32 (average modern-day Saharan albedo). The ozone distribution is prescribed using a dynamic approach (*184*) in which ozone is dynamically coupled to the model tropopause height with constant values for the troposphere [0.02 parts per million (ppm)], tropopause (0.2 ppm), and stratosphere (5.5 ppm). The model includes a recent update that modifies cloud condensation nuclei density and cloud droplet effective radius to better represent high-latitude warmth as highlighted by proxy observations using the methods of Sagoo *et al.* (*185*) and Kiehl and Shields (*186*), allowing a more representative pole-to-Equator temperature gradient to develop under warmer climates of the past, while still producing a skillful pre-industrial climate. Other external forcings (for example, orbital parameters and aerosols) were held at pre-industrial values to isolate paleogeographic/*P*co_2_ controls on climate [see Farnsworth *et al.* (*187*)] and Valdes *et al.* (*188*) for further details]. Each experiment was integrated for multi-millennial durations to ensure full model equilibrium (i.e., >6000 model years). Convergence diagnostics at the end of integrations satisfied standard criteria: globally and volume-integrated mean ocean-temperature drift < ∼1°C per 1000 years, surface-air-temperature drift < ∼0.3°C per 1000 years, and a top-of-atmosphere net energy imbalance < ∼0.25 W m^−2^ (100-year mean). Climatological means were computed from the final 100 years of each time interval.

For ENM projections, we extracted gridded near-surface air temperature and precipitation from each time slice and converted monthly fields to standard bioclimatic derivatives. Consistent with our variable-screening and collinearity tests, the final explanatory set comprised temperature of the warm months (wmm), temperature of the cold months (cmm), precipitation of the wet months (wetmon), and precipitation of the dry months (drymon). To ensure spatial consistency between rotated fossil occurrences and climate fields, we used the same plate configurations, coastlines, and land-sea masks that underpin the paleogeographic boundary conditions; fossil localities were rotated into paleocoordinates (*178*) on those plate models before climate extraction. Each ENM projection was matched to the midpoint age of its geological sub-stage/bin (5-Myr spacing across the Phanerozoic series used here). All paleoclimate rasters were bilinearly regridded to a common 1° grid and masked to emergent land for the corresponding slice. Following Chiarenza *et al.* (*16*), we did not apply modern bias correction (for example, delta-method adjustments) to deep-time simulations to avoid distorting modeled paleosignals. Instead, predictors were standardized within slice, and we used MESS/environmental-range diagnostics during projection to identify and limit extrapolation into non-analog climates (with clamping enabled where appropriate). These steps provide uniform spatial resolution and paleogeographic alignment through time while preserving the internal structure of the HadCM3L fields used to derive the bioclimatic variables.

### Environmental variable choice

We processed paleoclimate layers in R using terra (*189*), RNetCDF (for NetCDF access), and rgplates (*190*) (for plate-rotation utilities and coastlines from the GPlates web service). Time-slice stacks of gridded variables (ASCII/NetCDF) were imported as SpatRaster objects and harmonized to a common geodetic CRS (WGS84; EPSG:4326). For each slice, we reconstructed coastlines at the target age with rgplates [PALEOMAP plate model; Scotese (*177*)], transformed them to WGS84, and converted to vector masks. Climate rasters were then clipped to emergent land via terra::mask. Where needed, layers were bilinearly resampled to a common grid before export for ENM fitting and projection. Visual quality assurance and quality control consisted of plotting the reconstructed coastlines against masked rasters for each slice to confirm alignment and coastline fidelity. To minimize overfitting from correlated predictors, we computed pairwise Pearson correlations on the masked, slice-specific stacks using terra::layerCor, inspected correlation heatmaps (corrplot), and performed hierarchical clustering on |*r*| distances (hclust on 1 − |*r*|). Following the thresholding approach we apply in deep-time ENMs, we cut the dendrogram at |*r*| = 0.75, retaining at most one variable per high-correlation cluster. Applying this procedure across slices and enforcing a single, time-invariant predictor set for comparability, we selected four quarter-based extremes as explanatory predictors: wmm (mean temperature of the warmest quarter), cmm (mean temperature of the coldest quarter), wetmon (total precipitation of the wettest quarter), and drymon (total precipitation of the driest quarter). This final set captures seasonal thermal and hydric bounds most relevant to crocodylian physiology (*191*–*194*) while reducing redundancy with annual aggregates (*15*, *16*, *27*, *172*, *179*). All rasters used for ENMs were written as harmonized ASCII grids and masked consistently using the slice-specific coastlines and paleogeography used throughout the study.

### Paleoclimate models of K/Pg scenarios

To capture short-lived forcing around the K/Pg while keeping full consistency with the rest of our deep-time framework, we used the same coupled AOGCM (HadCM3L-M2.1aD) at 3.75° × 2.5° resolution with 19 atmospheric and 20 ocean levels, B-grid numerics, zero-layer sea ice, MOSES-2.1a land surface, and dynamic vegetation (TRIFFID; five plant functional types) as above. The ∼5-Myr temporal resolution of our long-term ENM and PPGM projections cannot resolve the short-lived (decadal-to-centennial) climatic perturbations associated with the Chicxulub-impact event. To assess pseudosuchian habitat suitability across these transient (i.e., dynamically evolving through time) forcings, we performed a separate suite of coupled AOGCM transient simulations using the same HadCM3L-M2.1aD model configuration at 3.75° × 2.5° resolution, following the approach of Chiarenza *et al.* (*27*). All K/Pg simulations share Maastrichtian stage-specific boundary conditions (topography, bathymetry, coastlines, and land-sea mask) and a Maastrichtian solar constant; baseline CO_2_ is 560 ppm (≈2× pre-industrial), within proxy ranges (*182*) for the Late Cretaceous. We first produced a fully equilibrated Maastrichtian control (10,122 model years) and then branched transient experiments from this baseline, analyzing 50-year climatologies from the end of each run.

Impact-winter forcing was represented in two ways, following the study system in Chiarenza *et al.* (*27*). First, we prescribed stratospheric sulfate aerosol burdens by scaling the observed optical depths from the 1991 Pinatubo eruption to 50× and 100× at 0.55 μm, with radiative effects applied across 0.2 to 10 μm and a fixed aerosol size distribution. Aerosols (optical depth) were injected into the stratosphere in four geographic bands starting in year 40 of 200-year integrations and allowed to decay with a 6-year residence time, reflecting enhanced stratification under surface cooling; no background Maastrichtian aerosol source was imposed outside this window. To represent the surface consequences of widespread fallout, we also ran variants with a continent-scale ash blanket over North America (≈15 to 67.5° N, 116.25 to 26.25° W) that modifies land surface albedo and infiltration; to avoid an unrealistically sterile surface, we permitted a small fractional cover of fast-colonizing C3 vegetation within the blanket using TRIFFID defaults. Second, to bracket uncertainty in shortwave attenuation by sulfate haze, we imposed idealized solar-dimming experiments that reduced top-of-atmosphere insolation by 5, 10, 15, and 20% for 200 years under the 2 × CO_2_ baseline, which approximates the net radiative effect of large sulfate loads and, at the upper end, provides an impact analog. The solar-dimming magnitudes represent sustained multi-year to decadal reductions in surface insolation, not instantaneous or monthly maxima, and bracket the range of published estimates for Chicxulub ejecta radiative effects (*27*, *28*, *195*, *196*).

Longer-lived volcanogenic forcing was represented with CO_2_ enrichment experiments designed to test whether Deccan degassing could offset aerosol-driven cooling and broaden suitable climates. We ran 1000-year integrations in which CO_2_ was stepped from 2× to 4× and to 6× the pre-industrial value (280 ppm) at *t* = 0 (instantaneous step chosen for computational tractability and to sample the upper end of published estimates), and we paired these with 200-year transients that combined CO_2_ increases with aerosol and/or ash forcings. Across all K/Pg runs, we extracted near-surface air temperature (1.5 m) and total precipitation and, where available, monthly extremes (for example, warmest/coldest month temperature: wmm/cmm; wettest/driest month precipitation: wetmm/drymm). These fields were bilinearly regridded to 1° and masked to the Maastrichtian land mask, then harmonized with our paleogeographic rotation so that the same plate configuration used to place fossil occurrences also underpinned the climate surfaces supplied to ENM hindcasts and PPGM-based projections. Orbital parameters and other minor forcings were held at pre-industrial values for all K/Pg experiments to isolate the effects of aerosols, ash, and CO_2_.

### Ecological niche modeling

We implemented an ensemble ENM workflow in R with biomod2 4.2-6 (*197*) [following and updating the protocol in Chiarenza *et al.* (*16*)]. Presence records (filtered to one per climate grid cell; see the Occurrence data assembly and filtering section) were paired with random pseudo-absences constrained to emergent land within each target slice. For each modeling run, we drew 1000 pseudo-absences and repeated the draw four times to propagate background uncertainty. Models were fit across a standard algorithm panel spanning both regression- and machine-learning families: GLM, GAM, MARS (earth), FDA (mda), Random Forest, Maxent (via maxnet), XGBoost, and SRE/BIOCLIM. Hyperparameters were tuned using biomod2 automated strategies (including the “bigboss” and “tuned” options), with variable importance computed per algorithm (permutation importance, three repeats). To evaluate discrimination, we used true skill statistic (TSS) as the primary metric and ROC/AUC as a secondary diagnostic. Models were trained and tested under repeated random cross-validation (80/20 splits, two repeats). In addition, we generated *k*-fold (*k* = 3) and geographically stratified (twofold) cross-validation partitions to check for spatial leakage; results from these schemes were used as sensitivity analyses and did not change the main conclusions. We retained only algorithm runs with TSS ≥ 0.70 for ensemble construction. Consensus predictions were built with BIOMOD_EnsembleModeling, using EMwmean (TSS-weighted mean) as our primary ensemble and reporting EMmean and EMmedian as unweighted comparators. We also calculated EMcv and EMci (95% confidence) to summarize cross-validation variability. For each slice, ensembles were projected to paleoclimate covariates with identical predictor definitions (wmm, cmm, wetmon, drymon), and we generated a clamping mask to flag extrapolation beyond the observed environmental range. Where binary outputs were required (e.g., areal calculations), we applied algorithm-specific TSS-optimized thresholds and combined them under the chosen ensemble rule. To quantify spatiotemporal signals, ensemble suitability was summarized per slice as (i) latitudinal distributions of suitability and (ii) total suitable area (binary). We report ensemble medians and interquartile ranges across cross-validation replicates and algorithms to characterize uncertainty, using medians to quantify niche occupation. Response curves and variable-importance profiles were inspected to ensure that seasonal thermal (wmm, cmm) and precipitation (wetmon, drymon) limits, rather than collinear annual aggregates, drove predictions, consistent with physiological expectations for crocodylians (*191*–*194*) and the deep-time ENM practice for extinct archosaurs in Chiarenza *et al.* (*16*, *172*, *173*, *179*) and Foffa *et al.* (*43*). To characterize spatial dynamics of suitability through time, we calculated the centroid of habitat suitability for each slice (*15*, *16*) using the TSS-weighted ensemble mean (EMwmean) as our primary continuous surface and repeating calculations on binary ensembles (LTP and MaxSSS) as a sensitivity check. Because grid-cell area varies with latitude, suitability rasters were first reprojected to an equal-area coordinate system (Mollweide), and centroids were computed with cell-area weights; for continuous ensembles, weights were the product of suitability and cell area, and for binary ensembles, weights were cell area for suitable cells and zero otherwise. To avoid artifacts near the 180° meridian, centroid positions were derived using spherical averaging of weighted unit vectors and then converted back to geographic coordinates. For interpretability, we additionally summarized the area-weighted mean latitude of suitability and its interquartile range, and we quantified inter-slice shifts as great-circle (Haversine) distances between consecutive centroids. All centroid metrics were computed per cross-validation replicate and then aggregated (median, interquartile range), providing conservative, area-aware estimates of the spatiotemporal “center of mass” of suitable climates.

### PPGM of climatic envelopes

Phylogenetic comparative methods were applied to species-level climatic niche summaries following established practice for treating niche parameters as continuous traits (*150*, *198*). Although ecological niches are not heritable phenotypic traits in the strict sense for which comparative methods were originally developed, climatic niche characteristics are constrained by heritable physiological tolerances, a case of extended phenotype [sensu Dawkins (*199*)], and can therefore be analyzed in a phylogenetic framework. Here, MAT and MAP were treated as continuous traits and analyzed using phylogenetic relationships and alternative evolutionary models, following the same logic commonly applied to traits such as body mass or limb proportions (*150*). This framework allows us to compare BM, EB, and OU models of niche evolution, reconstruct ancestral climatic values, visualize trait changes through time, and identify the timing and location of major niche shifts. We implemented these analyses in R using contMap and PPGM projections. Similar approaches have been applied across a range of vertebrate groups, including primates (*200*), squamates (*163*, *201*), and archosaurs, including birds (*13*, *202*). We further distinguish between the realized niche, inferred from observed occurrence data, and the fundamental niche, representing the broader set of conditions a species can physiologically tolerate before filters due to biotic factors (e.g., dispersal or competition) (*150*, *203*, *204*). Accordingly, ENMs calibrated on present-day occurrences primarily estimate realized climatic niches, whereas phylogenetically informed PPGM reconstructions provide a complementary estimate of ancestral climatic tolerances.

We reconstructed the evolutionary history of the pseudosuchian climatic envelope and generated phylogenetically informed spatial predictions by producing PaleoPhyloGeographic Models [PPGM (*31*)] in the PPGM R package v1.1 (*205*). These models use a phylogenetic comparative framework to estimate changes in climate tolerances through time. For each terminal taxon (both extinct and extant) with adequate (=recorded coordinates) data, we summarized climatic traits from the ENM-ready occurrence × predictor table by extracting the four explanatory variables used throughout (MAT, MAP, temperature of the warm months, wmm; temperature of the cold months, cmm; precipitation of the wet months, wetmon; precipitation of the dry months, drymon) and then selecting for PPGM computation the least explanatory (two) (*31*) variables that mostly affected pseudosuchian distribution (MAT and MAP). For each taxon and variable, we computed the minimum and maximum (envelope bounds) and a measure of central tendency (median).

We used the comprehensive time-calibrated phylogeny of Pseudosuchia published by Payne *et al.* (*175*), which was generated using a tip-dated fossilized birth–death (FBD) framework and provides branch lengths in millions of years for all included taxa. Species names in our dataset were matched to those in the phylogeny using the phylo_check() function in palaeoverse (*178*). The original phylogeny contained 534 taxa and was then pruned to retain only taxa with available climatic data, and branch lengths were handled in a time-calibrated framework using paleotree::timePaleoPhy. Marine-obligate lineages were excluded from terrestrial suitability analyses (e.g., strictly pelagic thalattosuchians and broader tethysuchians; Fig. 1A); brackish/estuarine occurrences were retained when depositional context indicated a terrestrial-fresh water association consistent with our paleoclimate layers, obtaining a total of 402 tips. Before calibration, we harmonized tip labels and ages, corrected any FAD < LAD inconsistencies using the phylo_check function of palaeoverse (*178*), and ensured non-negative ages.

We compared alternative models of climatic trait evolution, BM, EB, and OU (*136*), using AICc (Akaike information criterion corrected for finite sample size) as the primary model selection criterion, with ΔAICc >2 considered meaningful support. To quantify the strength of phylogenetic signal in each climatic variable independently of explicit evolutionary model choice, we estimated two complementary metrics. Pagel’s λ (*5*) rescales internal branch lengths to assess whether trait covariance across tips conforms to BM expectations: λ ≈ 1 indicates strong phylogenetic dependence consistent with BM; λ ≈ 0 indicates phylogenetic independence. Blomberg’s *K* (*206*) compares the magnitude of among-lineage variance relative to the BM expectation: *K* = 1 equals the BM prediction; *K* < 1 indicates that within-clade variance is compressed below the BM expectation, consistent with multi-optima OU dynamics; *K* > 1 indicates stronger-than-BM phylogenetic clustering. The high-λ/low-*K* combination is the empirical fingerprint of multi-optima OU evolution (*10*). Both metrics were estimated by maximum likelihood using the time-calibrated tree and species-level climatic medians, and statistical support for λ was assessed by likelihood ratio tests comparing the fitted λ model against constrained models with λ fixed at 0 and 1, respectively. All variables showed significant phylogenetic signal (λ significantly greater than 0, *K* significantly less than 1), supporting the use of phylogenetically informed ancestral reconstructions and downstream paleogeographic projections (*207*).

OU provided the best fit for both MAT and MAP (Supplementary Data). OU parameters (attraction rate α, long-run optimum θ, and stochastic variance σ^2^) were estimated via likelihood optimization using fitContinuous [geiger package (*208*)] with broad but finite parameter bounds (α ∈ [0, 10^5^], σ^2^ ∈ [0, 10^6^]). For MAT, the fitted parameters are α = 0.0174 (95% CI: 0.0123 to 0.0235), θ = 23.1°C, σ^2^ = 1.320 (95% CI: 1.077 to 1.660), giving a phylogenetic half-life (ln 2/α) of 39.9 Ma (95% CI: 29.5 to 56.4 Ma). For MAP (analyzed on a log_10_-transformed scale to satisfy the normality assumption of the OU likelihood and stabilize variance), α = 0.078, θ = 1929 mm, σ^2^ = 0.0056, giving a half-life of 8.9 Ma (95% CI: 4.2 to 14.8 Ma). The stationary variance (σ^2^/2α) (the long-run trait dispersion expected under the fitted OU) is 37.9°C^2^ for MAT and 0.036 log_10_(mm)^2^ for MAP, both substantially lower than BM would produce over the clade age of 252 Ma. We emphasize that the OU model is a statistical description of the macroevolutionary pattern (*136*): Its OU regime was used to estimate node states for all variables on the time-scaled tree, with BM models retained as sensitivity analyses and propagated through to spatial projections.

### Multi-regime OU analyses: Bayou and SURFACE

To test for shifts in OU optima (θ) across the pseudosuchian phylogeny without prespecifying boundary positions, we applied two complementary free-search multi-regime OU frameworks: bayou v2.2 (*209*) and SURFACE (*210*). These frameworks were applied to MAT and MAP independently and constitute the primary inference tools for regime-shift detection; their results are reported in the Results section and interpreted in the Discussion. A prespecified boundary comparison using OUwie (*211*, *212*) was conducted as a pilot analysis but eventually discarded owing to a tip-imbalance artifact. We tested whether the multi-model comparison analyses captured any significant transition in θ without a priori assumptions on when these transitions might have been located, and then, a posteriori, test whether these shifts coincided with important mass extinction or suggested biotic crises caused by geologic hyperthermals in the last half of the Phanerozoic [T/J (*147*), Jenkyns Oceanic Anoxic Event (*138*), Jurassic/Cretaceous (*71*), C/T (*213*), K/Pg (*214*), Paleogene-Eocene Thermal Maximum (*215*), E/O (*216*), Middle Miocene Climatic Optimum (*217*)].

For this reason, the greater reliability of the bayou implementation stand on its reversible-jump Markov chain Monte Carlo (RJMCMC) to infer the number, location, and magnitude of θ shifts as random variables integrated over phylogenetic uncertainty, without prespecifying any boundaries. Two independent MCMC chains were run for 2,000,000 generations each for both MAT and MAP (30% burn-in; post-burn-in samples combined into a single posterior). Convergence was assessed using the Gelman-Rubin statistic (*R̂*) and effective sample size (ESS) for log-likelihood and the number of shifts (*k*). For MAT, *R̂* ≤ 1.041 for all parameters and ESS(*k*) = 1143; for MAP, *R̂* ≤ 1.009 and ESS(*k*) = 1076. The median number of shifts in the post-burn-in posterior was 11 for MAT (median lnL = −1221.66). Branches with marginal posterior probability (PP) ≥ 0.50 for a θ shift were identified as high-support shift locations (PP ≥ 0.10 and ≥ 0.30 also shown for context; Fig. 5, D and E). Shifts were assigned to geological boundaries when their age fell within ±5 Myr of a boundary; a root-adjacent shift subtending >95% of taxa was excluded from biological interpretation as a baseline-anchoring artifact. A boundary was considered robustly supported only where both bayou and SURFACE converged on a shift in the same time window.

SURFACE (*210*) implements a stepwise AICc-guided forward-backward search for multi-regime Hansen models. The forward phase adds regime shifts wherever ΔAICc improves by the threshold amount; the backward phase collapses regimes sharing statistically indistinguishable θ values to detect convergent evolution. To harmonize the SURFACE analysis across variables and avoid overfitting on the large (402 tips) tree, we applied a standardized protocol: tree rescaled to unit height, trait z-standardized before analysis, AICc improvement threshold ≥6 units (more conservative than the default of 2), and a hard cap of 15 forward steps. Only shifts placed on internal nodes, representing clade-level transitions rather than single-lineage idiosyncrasies, were retained for biological interpretation (*210*). Terminal single-tip shifts were recorded but excluded from the main results. For MAT, SURFACE identified 15 shifts collapsing to six adaptive zones (AICc = 902.84); for MAP, 15 shifts collapsing to seven adaptive zones (AICc = 931.57). The internal-node shifts for MAT fall within the C/T window (∼94.9 Ma) and near the PETM (∼51.3 Ma); the latter received negligible bayou support (PP ≈ 0) and is treated as a stepwise-ML artifact (see Results).

Ancestral state reconstructions for visualization (contMap; Fig. 5, A and C) were performed using phytools under the best-supported single-regime OU model, generating continuous ancestral state estimates at all nodes and along all branches. Phenograms (Fig. 5, B and D) were produced with a revised plotTraitGram function that fixes climatic *x*-axis limits for comparability across variables and optionally plots fossil climatic points (minimum/maximum age brackets). The bayou shift posterior probabilities through time (Fig. 5, D and E) were computed by binning branch midpoint ages into 5-Myr windows and summing PP across all branches within each window and then overlaid with the eight a priori geological boundary reference lines (T/J, Jenkyns Oceanic Anozic Event, J/K, C/T, K/Pg, PETM, E/O, and MMCO).

Following Lawing *et al.* (*163*), a pixel is classified as suitable for a given node if, for each selected climatic variable, the pixel’s value falls within the node’s reconstructed min-max envelope. We implemented both (i) a continuous variant, in which pixel weights are proportional to proximity to the node’s central tendency within the envelope, and (ii) a binary variant (inside/outside envelope). Unless noted otherwise, maps reported in the main text use the continuous definition for direct comparability with ENM maps. Node envelopes were projected to paleoclimate surfaces for each time slice. We incorporated our global paleoclimate rasters in the PPGM using the “paleoclimateUser” pathway in the PPGMConsensus function, passing (i) a list of slice-specific rasters reprojected to an equal-area CRS and harmonized to the climatic raster stack, and (ii) a matching “layerAge” vector (250 to 0 Ma, 5-Myr spacing) to align reconstructions to slices. To guard against spurious suitability in non-analog environments, we computed MESS maps with “PPGMMESS”; regions where any variable fell outside the training range were flagged and reported alongside MESS diagnostics (*163*). All scripts, occurrences, and age-keyed paleoclimate stacks (layerAge) in R-readable format are available at this DOI: 10.5281/zenodo.18354822.

## Supplementary Materials

### This PDF file includes:

Supplementary Text Table S1 References
