# Supplementary material for: Climatic niche conservatism shapes the macroevolution, extinction, and survivorship of crocodile-line archosaurs
Source: Sci Adv. 2026 Oct 21;12(43):eaeg2217. doi: 10.1126/sciadv.aeg2217 (PMC13685984; doi:10.1126/sciadv.aeg2217)
Supplement: Supplementary file 1 [file sciadv.aeg2217_sm.pdf]

Science Advances
AAAS

Supplementary Materials for

Climatic niche conservatism shapes the macroevolution, extinction, and survivorship of crocodile-line archosaurs

Alfio Alessandro Chiarenza *et al.*

Corresponding author: Alfio Alessandro Chiarenza, a.chiarenza15@gmail.com, alfio.chiarenza@ucl.ac.uk

Sci. Adv. **12**, eaeg2217 (2026)
DOI: 10.1126/sciadv.aeg2217

This PDF file includes:

Supplementary Text
Table S1
References

Supplementary Information

Extended comparisons between modelling outputs and the pseudosuchian fossil record, with future occurrence predictions

Body fossils of early pseudosuchians are scarce before the Late Triassic, but ichnological evidence like chirotheriid footprints attributable to crurotarsan-grade archosauriforms extends the record back into the Early–Middle Triassic and points to a low-latitude origins for the group(*32*, *33*). Extensive chirotheriid trackways from the Alps and adjacent regions such as *Isochirotherium* tracks at Vieux Emosson (southwestern Switzerland) and Lower Triassic archosauriform tracks from the Western Alps together with rich assemblages from the Germanic Basin and Iberia, document widespread archosauriform presence well before the Carnian–Norian body-fossil peak and help bridge gaps in the skeletal record(*34*–*38*). While trackmaker assignment and fine biochronology carry acknowledged uncertainties, the ichnological signal indicates an early, tropical–subtropical presence of pseudosuchian-line archosauriforms, broadly consistent with proposals of a low-latitude Gondwanan origin for the clade(*39*). The empirical record broadly aligns with a Late Triassic pseudosuchian belt (Fig. 4J, 5J) at mid–low palaeolatitudes, with dense Carnian–Norian fossil assemblages in the Chinle Group of the southwestern United States(*47*–*49*) and across southern South America in the Ischigualasto–Los Colorados and Santa Maria successions(220–222). Parallel occurrences in Laurasia (Keuper/Krasiejów of Germany–Poland(*50*–*52*)) and eastern Europe/Asia (Don–Volga and Siberian basins(53, 223)), plus northern China(45, 46), outline a quasi-continuous tropical–palaeotemperate corridor more extensively modelled by our projections than in the current fossil record. By contrast, conspicuous gaps remain for Carnian–Norian non-marine pseudosuchians in north-eastern and central–eastern Africa and the Arabian Peninsula: during this early window of crocodilian-line archosaur evolution, our PPGM and ENM models indicate strong suitability across these regions, areas that remain largely undersampled. Isolated occurrences in adjacent regions (for example, phytosaurs in Turkey(224)), may contribute to (and partially) validate the modelled suitability signal in this region. We therefore predict recoverable material in these areas, whose localities match modelled, but yet unsampled, corridors. Within southern South America, documented rauisuchians, early crocodylomorphs(225–227) and aetosaurs(228, 229), meet expectations for a well-connected belt, whereas southern Africa yields rarer but stratigraphically constrained material in the lower Elliot Formation (mid-Norian–Rhaetian(230)), collectively matching the inferred palaeoequatorial corridors towards higher latitudes of this Late Triassic interval. We interpret these ’waves’ of suitability expansion into dispersal corridors as the geographic imprints of humidity increases that ameliorated arid conditions, establishing dispersal corridors along a broad palaeoequatorial belt spanning central–western Africa and extending towards South America, plausibly linked to the Carnian Pluvial Event(*42*–*44*). These predictions suggest that targeted lower Elliot Formation-

age surveys in other southern Gondwanan localities will be particularly rewarding of crocodylomorph occurrences(*40*) in the future.

Through the Rhaetian and into the Hettangian, modelled suitability is broadly low and geographically restricted, concentrated in equatorial coastal belts, with gradual reduction in continental interiors relative to the Norian peak. No sharp minimum is observed at the boundary itself. Recovery begins within the Early Jurassic, rising from the Sinemurian and especially through the Pliensbachian–Toarcian. The fossil record broadly matches this pattern (few Hettangian–early Sinemurian localities, richer Pliensbachian–Toarcian assemblages). Any remaining mismatch reflects that the modelled early recovery outpaces the available fossils, likely due to preservational or sampling gaps. Targeted work should therefore focus on (i) Hettangian equatorial coastal–fluvial refugia predicted by the models and (ii) earliest Sinemurian basins along the inferred dispersal corridors.

Terrestrial crocodylomorphs become more abundant in the Middle–Late Jurassic of northern Laurasia (Fig. 4H), including the UK (e.g. Isle of Skye(*57*)) and Germany (Solnhofen–Kelheim limestones(*58*)). These occurrences document small-bodied non-marine neosuchians in near-coastal to lagoonal settings, and confirm sustained mid–high latitude occupancy across the European region(*57*). In western Laurasia, the Morrison Formation preserves both small terrestrial crocodyliforms (e.g. *Fruitachampsa*, *Macelognathus*) and neosuchians in fluvial–lacustrine systems (e.g. *Eutretauranosuchus*, *Amphicotylus*), reinforcing continued occupation of North American mid-latitudes and matching these modelled mid to high-latitude windows. In eastern Laurasia, multiple Late Jurassic shartegosuchids (e.g. *Shartegosuchus*, *Nominosuchus*) demonstrate an independent Asiatic record of small terrestrial crocodyliforms that aligns with modelled high-latitude windows in this palaeocontinent(*54*). Our models also predict notable geographic outliers that match the fossil record in China (Lufeng(*55*)), India-Madagascar(231), and South Africa(*62*). Both ENM- and PPGM-based spatial predictions reconstruct high suitability across many of the intervening areas, with especially elevated suitability across central–eastern Africa, implying a near-continuous eastern Gondwanan corridor between the southern African and Asian outliers. This eastern Gondwana area though is much more weakly sampled (Fig. 1C): Australia has no confidently documented Jurassic terrestrial crocodylomorph body fossils (the Australian eusuchian *Isisfordia* marks an Early–mid-Cretaceous appearance(*64*); Fig. 4G, 5G).

Antarctica presently lacks confirmed Jurassic terrestrial crocodylomorph body fossils; in India, putative Kota Formation material remains indeterminate(65, 232). In Madagascar notosuchians are reported(233). Both ENM and PPGM projections, however, indicate high Middle–Late Jurassic suitability across central–eastern Africa, implying a near-continuous eastern Gondwanan corridor linking southern Africa and Asia (Fig. 4H, 5H). Predicted poleward windows into Antarctica and Australia remain untested because these regions are undersampled for Middle–Late Jurassic terrestrial vertebrates. We therefore anticipate future discoveries of small-bodied terrestrial

crocodyliforms, most plausibly shartegosuchid-grade forms or basal notosuchians and neosuchians in Indian Gondwana and Antarctic strata, especially lacustrine to marginal-marine settings and pedogenically overprinted floodplains that often yield microvertebrates.

With suitability remaining stable across the Jurassic–Cretaceous boundary in our models (Fig. 4H, 5H), the Berriasian–Valanginian Purbeck–Wealden complexes(*66*, *67*) provide the clearest body-fossil signal (Fig. 1D), yielding a rich pseudosuchian assemblage whereas the Tuchengzi Formation in northern China (Tithonian–Berriasian) remains dominated by crocodylian ichnites(*68*, *69*). We predict diagnostic crocodyliform body fossils at, or just above, the boundary within Tuchengzi Formation-equivalent redbeds of Northern China and Mongolia, where taphonomic windows comparable to Purbeck–Wealden assemblages are present but less intensively surveyed(*70*). During the Aptian–Albian, the North American record clusters along the Gulf–Western Interior margin(*80*, *81*), with interior occurrences scarce, coherent with a modelled contraction and implying that rare neosuchians from continental interiors should be sought in humid interfluves (e.g. Antlers Formation(*82*)) and crevasse-splay facies rather than main-channel sand bodies. Along the southern Tethyan belt (Fig. 1D), the Elrhaz Formation of Niger yields non-marine crocodyliforms(234), and Australia's Eromanga–Winton system preserves *Isisfordia duncani*(*64*), supporting an equatorial–subtropical corridor from Africa to eastern Gondwana; we expect further pseudosuchian diversity in central African basins (Congo, Benue) at these horizons.

Despite modelled high suitability (Fig. 4G, 5G), the terrestrial Cenomanian record remains comparatively sparse (Fig. 1D). This trough is plausibly preservational: the Cenomanian–Turonian sea-level highstand(73, 235) favoured marginal-marine deposition at the expense of extensive inland floodplains, reducing both accommodation space and the preservation potential of continental crocodylomorphs(*74*). Where Cenomanian continental strata are exposed, they are geographically clustered along the Western Interior Basin (WIB) within microsites(*75*) or Tethyan–northcentral Gondwana margins that yield only a handful of terrestrial/freshwater forms (e.g. peirosaurids in the Kem Kem Group; *Coringasuchus* in the Alcântara Formation(*76*–*78*)), and on the southeastern Gondwanan margin (Winton Formation, Australia(*79*)). By contrast, deeper inland basins that our models identify as suitable remain weakly sampled or poorly dated. Additional age uncertainty (Aptian–Albian vs. Cenomanian–Turonian binning) further depresses a potentially high Cenomanian diversity. These caveats explain the apparent mismatch between high modelled suitability and limited non-marine occurrences. We therefore predict early-diverging neosuchians at Cenomanian–Turonian levels in the WIB(236), where suitability is elevated but latitudinally contracted, and notosuchians in Arabian Plate basins (Levant, Mesopotamian foreland(237)), where modelled suitability is high.

While sampling is too poor for meaningful comparisons in the Coniacian–Santonian, from the Campanian to the Maastrichtian crocodyliform occurrences concentrate along latitudinally wide sectors of the Western Interior Seaway (WIS; e.g.,

Deinosuchus, *Brachychampsa*(*83*, *84*)), reaching ~67° N. However, they were far from confined to North America: in Europe, neosuchians and notosuchians are widespread across the Ibero-Armorican, Adriadic-Dinaric islands and the Transylvanian Basin(87, 238–241); in Asia, gobiosuchids/shartegosuchids and neosuchians occur in Campanian–Maastrichtian deposits of Mongolia and northern China (e.g., *Gobiosuchus*, *Shamosuchus*(*59*, *90*)); in South America, the Bauru Group yields a diverse notosuchian–neosuchian fauna (e.g., *Baurusuchus*, *Stratiotosuchus*, *Mariliasuchus*, *Peirosaurus*(91, 92, 242, 243)); and in Madagascar, Maastrichtian forms include *Simosuchus* and *Mahajangasuchus*(93, 94). Taken together, these records indicate a broadly circumtropical–subtropical to temperate distribution in the latest Cretaceous spanning the Americas, Europe, parts of Asia and the northern Africa–Arabia margins, consistent with the geographic and latitudinal pattern of suitable habitats predicted by our PPGM–ENM models. Due to global high suitability in palaeo-temperate–equatorial regions, we anticipate additional Maastrichtian material in Indian intertrappean beds(*27*, *95*, *96*) and in equatorial South America(244). Across the K/Pg boundary (Fig. 4E, 5E, Fig. 6), suitability persistence and rapid post-impact recovery is inferred from model projections and is reflected by Danian occurrences in the Gulf region, North and West Africa (dyrosaurids), and northern South America. We predict additional Danian freshwater crocodyliforms from fluvial–lacustrine successions in central–western Africa and northeastern Brazil, where continuous early Palaeocene sedimentation may capture low-latitude refugia within a tropical belt that was comparatively buffered from impact-winter cooling(*95*–*100*). Post-K/Pg recovery to broader latitudinal ranges appears delayed (Fig. 4E, 5E): although stable at tropical bands, suitability only returns to pre-extinction breadth over the first ~10 Ma of the Cenozoic.

In North America, this modelled range extension is consistent with Paleocene records from the Red Deer River, Canada, and the american Paskapoo Formation, including the Saxonella Locality and Birchwood(245, 246). In Asia, elevated suitability is consistent with late Paleocene assemblages from the south-western Gobi in China(247). In Europe, occurrences from Jeumont, France (the area associated with *Eosuchus lerichei*) and Belgium offer further support to northern Europe expansion of crocodylians in the Paleocene(248, 249). Fossil occurrences from the early Eocene (Ypresian) register a pronounced poleward extension (Fig. 1E) that mirrors modelled habitat-suitability expansion (Fig. 4D, 5D): high-latitude North American occurrences from the northern Great Plains/Rocky Mountains to Ellesmere Island(250), gavialoids in the North Sea–British basins (e.g., Isle of Sheppey), and rare Antarctic records on Seymour/Marambio Island collectively indicate widened climatic space during the Early Eocene Climatic Optimum(*101*, *105*, *106*). Additional high-latitude finds in the Wasatchian of North America(*106*) and at Sheppey(*107*) provide independent support for this poleward expansion of their habitat suitability into higher latitudes. Ypresian projections show a pronounced increase in suitability spanning both western and eastern Antarctica and extending into Australia, with smoother, more continuous gains across North America. These patterns are corroborated by relatively higher-latitude

occurrences in North America at White Butte/Turtle Valley and Swami's Point(251), by Cañadón Hondo and Bird Clay localities in Argentina(*102*), and by records from Tsagaan Khushuu in Mongolia(*103*). Additional Ypresian South American occurrences from Sierra Dorotea, Chile(*101*, *104*), also match this modelled expansion. Late Eocene (e.g. Bartonian) suitability at high latitudes (>50°) is supported by occurrences in both hemispheres, including Patagonia (*Eocaiman cavernensis* from *Notostylops* Beds(252)), the Antelope Creek locality in South Dakota(253), and by Seymour Island records (RV-8200 and RV-8405 sections) in Antarctica(*105*).

The observed Oligocene record conforms with the modelling of a cooler climate, impacting a contracting niche (Fig. 4C, 5C). In Europe, freshwater alligatoroids persist through the Rupelian–Chattian in Europe, with *Diplocynodon* documented in multiple basins, matching modelled temperate pockets around the Paratethys(*110*, *111*). In Afro-Arabia, gavialoids occur into the early Oligocene of the Fayum (*Eogavialis africanum;* see(*112*)), in line with sustained low-latitude suitability along the northern African margin. North America records early Oligocene alligatorids in continental successions and a Late Oligocene (~28–26 Ma) appearance of *Alligator* in the south-east, consistent with predicted gulfal/lowland refugia(113). In Asia, gavialoids continue across the Eocene–Oligocene boundary in south-east Asia (Krabi lignites, Thailand), and large crocodyloids occur in the Oligocene of the Bugti Hills (Pakistan), in agreement with modelled continuity along the northern Indian Ocean rim(108). By contrast, South America shows a pronounced sampling trough, with only scattered or fragmentary records(*115*), despite modelled suitability remaining comparatively high and stable. Antarctica lacks Oligocene crocodyliform body fossils, consistent with the low-latitude suitability inferred for this cooling interval. Australia maintains relatively higher levels, although contracting, of habitat suitability, a pattern confirmed by the fossil record of these regions(*114*).

Miocene models show crocodyliform suitable range expansion (Fig. 4B, 5B), likely due to the Mid-Miocene Climate Optimum (MMCO(*117*)), and is validated by a globally well-sampled fossil record (Fig. 1F). Through the Burdigalian and Langhian, models show a slight but geographically widespread increase in latitudinal extent, particularly in South America, Australia–New Zealand, and southern Africa, even as some continental interiors (e.g. parts of central Africa) experience habitat suitability losses. These patterns are corroborated by Burdigalian occurrences at Puerta del Diablo in Argentina(*118*) and in New Zealand (Croc Site L1, Mata Creek(*119*, *120*)), as well as by North American material from *Aletomeryx* Quarry(120). Langhian occurrences include Kleinfelder Farm in Canada(254) and several European sites such as the Hambach mine in Germany(116). Continued Langhian–Tortonian suitability in eastern North America at higher palaeotemperate latitudes is aligned with classic faunas from the Calvert Cliffs, Maryland(255), and from Cerro Aisol, Argentina(256), while Japanese Miocene occurrences from Fukui(*122*) confirm a high-latitude western Pacific presence. In Africa, Arrisdrift and the lower Orange River Valley yield *Crocodylus gariepensis* and closely referrable material(*123*, *124*), and further southern

Namibian occurrences at Auchas(257) reinforce the modelled southern African suitability through the MMCO. Additionally, fossil occurrences from New Zealand's Mata Creek(120) show that Southern Hemisphere temperate belts remained intermittently suitable as mid-Miocene warmth waxed and waned and suggests that renewed prospecting at such latitudes, particularly southern Africa's inland basins and New Zealand's lacustrine margins, should yield additional neosuchians that tracked MMCO warming.

Zanclean models indicate high suitability in regions with sparse but documented crocodylian diversity (Fig. 1G): for example in southern USA(127, 258), south-western South America(128), south-central Africa(129), India(130), and Australia(259), but also predict suitable climates in currently unsampled areas such as the north-western USA and parts of Europe (e.g. the UK and Iberian coastlines). PPGM outputs consistently recover broader high-latitude suitability than ENM, including Japan, New Zealand, and the southernmost South America. Consistent with these projections, Pleistocene–Holocene occurrences extend to latitudes higher than the present-day crocodylian range (e.g., Japan, northern China, South Africa, Maryland, New Caledonia, and eastern Mediterranean(131–135)). This signal is also reflected in pre-Industrial climate projections: both frameworks reproduce the modern tropical–equatorial distribution (Fig. 4A, 5A), but PPGM identifies additional, currently unfilled windows of high suitability in central–western Europe (Fig. 4A), whereas ENM predictions more closely track the realised modern range (Fig. 4A). A caveat is that our climate reconstructions operate at coarse temporal resolution (5 Ma) and therefore cannot capture local extirpations or range dynamics driven by shorter, high-frequency climatic oscillations (e.g., Quaternary glacial–interglacial cycles). These extra-tropical incursions, together with a late Neogene–Quaternary subfossil record that is likely more widespread than currently recognised, argue for targeted surveys of lowland basins with continuous deposition to test for short-lived range extensions that our 5 Ma climate surfaces cannot resolve. By contrast, the absence of crocodylian remains from continental Europe from the Pliocene onward most plausibly reflects genuine regional extirpation during cooler phases, rather than a sampling artefact.

Quantification of phylogenetic signal

To quantify the strength of phylogenetic signal in the two primary climatic variables analysed here, mean annual temperature (MAT) and mean annual precipitation (MAP), we estimated two complementary metrics. Pagel's λ (lambda)(5, 207, 260) rescales internal branches length to assess whether trait covariance conforms to Brownian motion (BM) expectations: expectations: $\lambda \approx 1$ indicates strong phylogenetic dependence; $\lambda \approx 0$ indicates phylogenetic independence. Blomberg's K(206) compares the magnitude of among-lineage variance relative to the BM expectation: $K = 1$ equals the BM prediction; $K < 1$ indicates within-clade variance compressed below

BM expectations, consistent with multi-optima OU dynamics; $K > 1$ indicates stronger-than-BM phylogenetic clustering. Both metrics were estimated by maximum likelihood using the time-calibrated tree and species-level climatic medians, and statistical support for λ was assessed by likelihood ratio tests comparing the fitted λ model against constrained models with λ fixed at 0 and 1 respectively (Table S1). For MAT, $\lambda = 0.99$ ($p < 0.001$) and $K = 0.334$ ($p < 0.01$); for MAP, $\lambda = 0.91$ ($p < 0.001$) and $K = 0.25$ ($p < 0.01$). The high-λ/low-K combination is the empirical fingerprint of multi-optima OU evolution (Table S1). All variables showed significant phylogenetic signal (λ significantly greater than 0; Table SI 1), supporting the use of phylogenetically informed ancestral reconstructions(207) and downstream palaeogeographic projections(*31*).

**Variable**	**λ**	**λ *p***	***K***	***Kp***
Mean annual temperature (MAT, °C)	0.9999	< 0.001	0.334	< 0.01
Mean annual precipitation (MAP, $\log_{10}$ mm yr^{-1})	0.91	< 0.001	0.25	< 0.01

Table S1. Pagel's λ and Blomberg's K for mean annual temperature (MAT) and mean annual precipitation (MAP), the two climatic variables analysed in the phylogenetic comparative methods. λ and K were estimated by maximum likelihood on the time-calibrated pseudosuchian phylogeny(175). MAP was $\log_{10}$-transformed prior to analysis. Statistical significance assessed by likelihood ratio tests (λ) and randomisation tests (K). The high-λ/low-K combination for both variables is diagnostic of multi-optima OU evolution (Cooper et al.(*10*)).

OUwie pilot analysis and tip-imbalance artefact

As an additional check on the multi-regime OU analyses, we ran a pre-specified boundary comparison using OUwie *(136, 206)* fitting two-regime OU models for each of eight geological boundaries in turn (T/J, Jenkyns OAE, J/K, C/T, K/Pg, PETM, E/O, MMCO), comparing single-regime OU against OUMA (independent α, σ^2, and θ per regime) by AICc. The Mid-Miocene Climatic Optimum (MMCO, ~16 Ma) was selected as the best boundary under OUMA for MAT. However, upon close inspection we identified this result as a numerical artefact, a commonly reported OUwie issue, of

severe tip imbalance and excluded the OUwie results entirely from the revised manuscript. The post-MMCO regime in our time-calibrated tree contains only 60 of 402 tips (~15% of the sample). The OUMA model has three independent parameters per regime (α, σ^2, and θ), giving six regime-specific parameters against three for single-regime OU. With only 60 tips to constrain three parameters for the post-MMCO regime, OUMA has substantial freedom to overfit the sparse post-boundary sample and achieve a spurious AICc improvement even in the absence of a genuine biological signal. This class of artefact is well documented for pre-specified boundary OU models on unbalanced trees(261, 262). To test whether this was indeed an artefact, we cross-validated the MMCO result against bayou, which places no boundaries a priori and infers shift locations as free parameters integrated over phylogenetic uncertainty. bayou returned a cumulative posterior probability of $PP < 0.05$ for any thermal-niche shift within ±5 Ma of the MMCO for MAT (Fig. 2D in the main text). The boundary-naive Bayesian free-search, which is not susceptible to pre-specification bias, does not corroborate the OUwie result, confirming it is a tip-imbalance artefact. We therefore rely exclusively on bayou and SURFACE as the primary inference frameworks, both of which must converge on a shift in the same time window for a boundary to be considered robustly supported. Under this framework, the sole robustly supported MAT boundary is the Cenomanian–Turonian (~93.9 Ma; bayou cumulative $PP = 0.61$; two convergent SURFACE internal-node shifts); the MMCO receives $PP < 0.05$ in bayou and no convergent SURFACE internal-node shift and is not reported as a biologically meaningful result.

Supplementary Data include the code, primary data, and raw outputs for the OU macroevolutionary, ENM and PPGM analyses. The ENM materials comprise the code, occurrence data (both raw and cleaned datasets prepared following the methodology described in the Methods of the main manuscript), environmental predictors (raster format), and modelling outputs from biomod2 and Maxent. The PPGM materials include the code, phylogenetic tree, occurrence data, climatic variable data, and raw outputs for the analyses, as described in the Methods of the main manuscript. All data, code and primary outputs of the analyses are available at this DOI: 10.5281/zenodo.18354822.

REFERENCES

1. J. A. Smith, E. M. Dowding, A. A. Abdelhady, P. Abondio, R. Araújo, T. Aze, M. A. Balisi, L. A. Buatois, H. Carvajal-Chitty, D. Chattopadhyay, M. Coiro, G. P. Dietl, C. G. Arango, C. Kevrekidis, J. Kimmig, A. M. Mychajliw, C. Pimiento, O. R. R. Fernández, K. M. Schroeder, R. C. M. Warnock, T.-R. Yang, M. Yasuhara, L. G. Akita, B. J. Allen, B. M. Anderson, J. Andréoletti, F. M. Archuby, G. A. Ballen, I. Bari, M. J. Benton, E. W. Bergh, L. Brambilla, A. Brombacher, Y. K. S. Chan, A. A. Chiarenza, T. Chinzorig, K. M. Coates, D. R. Cordie, M. Cortés-Sánchez, E. J. Cruz-Vega, J. D. Cybulski, K. De Baets, J. De Entrambasaguas, E. M. Dillon, A. Du, A. M. Dunhill, J. M. Erlandson, M.-B. Forel, W. J. Foster, T. A. Gates, A. Gavryushkina, M. K. Grace, H.-P. Grossart, P. Hänsel, P. G. Harnik, M. J. Hopkins, S. S. B. Hopkins, K. Hu, H.-H. M. Huang, R. B. Irmis, V. A. J. Jaques, X. A. Jenkins, A. M. Jukar, P. H. Kelley, R. G. Kihn, A. A. Klompmaker, Á. T. Kocsis, J. Kriwet, D. Lazarus, C.-C. Liao, C.-H. Lin, J. Louys, J. Lozano-Fernandez, M. C. Lozano-Francisco, J. A. Lueders-Dumont, M. E. Malvé, R. C. Martindale, I. Mazzini, G. Modenini, S. Mondal, M. Mondini, M. D. Monferran, L. P. A. Mulvey, K. Nanglu, J. M. T. Nguyen, R. Norris, A. O'Dea, A. L. Ollendorf, J. Orihuela, J. M. Pandolfi, T. Pereira, A. Piro, R. E. Plotnick, S. M. Plaza-Torres, A. Porto, A. Prieto-Márquez, S. W. Punyasena, T. B. Quental, N. B. Raja, V. Ranaivosoa, L. Ribas-Deulofeu, F. Rivals, V. J. Roden, A. Rosso, F. Saleh, R. B. Salvador, E. E. Saupe, S. Schneider, J. A. Sclafani, M. R. Smith, A. Souron, M. J. Steinbauer, M. Stewart, C. P. Tambussi, E. Thomas, E. Tschopp, T. Tütken, S. Varela, R. I. Vezzosi, A. Villaseñor, M. F. G. Weinkauf, L. E. Zanno, C. Zhang, Q. Zhao, W. Kiessling, Identifying the Big Questions in paleontology: A community-driven project. *Paleobiology* **51**, 408–431 (2025).

2. A. T. Peterson, J. Soberón, V. Sánchez-Cordero, Conservatism of ecological niches in evolutionary time. *Science* **285**, 1265–1267 (1999).

3. J. J. Wiens, C. H. Graham, Niche conservatism: Integrating evolution, ecology, and conservation biology. *Annu. Rev. Ecol. Evol. Syst.* **36**, 519–539 (2005).

4. G. G. Simpson, *Tempo and Mode in Evolution* (Columbia Univ. Press, 1944).

5. M. Pagel, Inferring the historical patterns of biological evolution. *Nature* **401**, 877–884 (1999).

6. C. Román-Palacios, J. J. Wiens, Recent responses to climate change reveal the drivers of species extinction and survival. *Proc. Natl. Acad. Sci. U.S.A.* **117**, 4211–4217 (2020).

7. A. D. Synodinos, R. Karnatak, C. A. Aguilar-Trigueros, P. Gras, T. Heger, D. Ionescu, S. Maaß, C. L. Musseau, G. Onandia, A. Planillo, L. Weiss, S. Wollrab, M. Ryo, The rate of environmental change as an important driver across scales in ecology. *Oikos* **2023**, e09616 (2023).

8. K. M. Bowgen, E. F. Kettel, S. H. M. Butchart, J. A. Carr, W. B. Foden, G. Magin, M. D. Morecroft, R. K. Smith, B. A. Stein, W. J. Sutherland, C. B. Thaxter, J. W. Pearce-Higgins, Conservation interventions can benefit species impacted by climate change. *Biol. Conserv.* **269**, 109524 (2022).

9. P. H. Harvey, M. D. Pagel, *The Comparative Method in Evolutionary Biology* (Oxford Univ. Press, 1991).

10. N. Cooper, W. Jetz, R. P. Freckleton, Phylogenetic comparative approaches for studying niche conservatism. *J. Evol. Biol.* **23**, 2529–2539 (2010).

11. J. B. Losos, Phylogenetic niche conservatism, phylogenetic signal and the relationship between phylogenetic relatedness and ecological similarity among species. *Ecol. Lett.* **11**, 995–1003 (2008).

12. J. J. Wiens, Commentary on Losos (2008): Niche conservatism déjà vu. *Ecol. Lett.* **11**, 1004–1005 (2008).

13. I. Quintero, M. A. Suchard, W. Jetz, Macroevolutionary dynamics of climatic niche space. *Proc. R Soc. B* **289**, 20220091 (2022).

14. J. J. Wiens, M. J. Donoghue, Historical biogeography, ecology and species richness. *Trends Ecol. Evol.* **19**, 639–644 (2004).

15. E. E. Saupe, A. Farnsworth, D. J. Lunt, N. Sagoo, K. V. Pham, D. J. Field, Climatic shifts drove major contractions in avian latitudinal distributions throughout the Cenozoic. *Proc. Natl. Acad. Sci. U.S.A.* **116**, 12895–12900 (2019).

16. A. A. Chiarenza, A. M. Waterson, D. N. Schmidt, P. J. Valdes, C. Yesson, P. A. Holroyd, M. E. Collinson, A. Farnsworth, D. B. Nicholson, S. Varela, P. M. Barrett, 100 million years of turtle paleoniche dynamics enable the prediction of latitudinal range shifts in a warming world. *Curr. Biol.* **33**, 109–121.e3 (2023).

17. K. D. Burke, J. W. Williams, M. A. Chandler, A. M. Haywood, D. J. Lunt, B. L. Otto-Bliesner, Pliocene and Eocene provide best analogs for near-future climates. *Proc. Natl. Acad. Sci. U.S.A.* **115**, 13288–13293 (2018).

18. J. T. Parrish, G. S. Soreghan, Sedimentary geology and the future of paleoclimate studies. *Sediment. Rec.* **11**, 4–10 (2013).

19. J. Y. Lee, IPCC AR6 WGI Chapter 4—Future global climate: Scenario-based projections and near-term information, in *Climate Change 2021: The Physical Science Basis*, V. P. Masson-Delmotte, A. Zhai, S. L. Pirani, C. Connors, S. Péan, N. Berger, Y. Caud, L. Chen, L. M. I. Goldfarb, M. Gomis, K. Huang, E. Leitzell, J. B. R. Lonnoy, T. K. Matthews, T. Maycock, O. Waterfield, R. Yelekçi, Yu, B. Zhou, Eds. (Cambridge Univ. Press, 2021), pp. 553–672.

20. P. J. Markwick, Fossil crocodilians as indicators of Late Cretaceous and Cenozoic climates: Implications for using palaeontological data in reconstructing palaeoclimate. *Palaeogeogr. Palaeoclimatol. Palaeoecol.* **137**, 205–271 (1998).

21. P. D. Mannion, R. B. J. Benson, M. T. Carrano, J. P. Tennant, J. Judd, R. J. Butler, Climate constrains the evolutionary history and biodiversity of crocodylians. *Nat. Commun.* **6**, 8438 (2015).

22. IUCN 2024: International Union for Conservation of Nature annual report (2024). https://iucn.org/resources/annual-reports/iucn-2024-international-union-conservation-nature-annual-report.

23. M. J. Benton, Scleromochlus taylori and the origin of dinosaurs and pterosaurs. *Phil. Trans. R. Soc. B* **354**, 1423–1446 (1999).

24. C. M. Holliday, E. R. Schachner, Dispatches from the age of crocodiles: New discoveries from ancient lineages. *Anat. Rec.* **305**, 2343–2352 (2022).

25. P. J. Markwick, Crocodilian diversity in space and time: The role of climate in paleoecology and its implication for understanding K/T extinctions. *Paleobiology* **24**, 470–497 (1998).

26. J. Cubo, M. V. A. Sena, P. Aubier, G. Houee, P. Claisse, M. G. Faure-Brac, R. Allain, R. C. L. P. Andrade, J. M. Sayão, G. R. Oliveira, Were Notosuchia (Pseudosuchia: Crocodylomorpha) warm-blooded? A palaeohistological analysis suggests ectothermy. *Biol. J. Linn. Soc.* **131**, 154–162 (2020).

27. A. A. Chiarenza, A. Farnsworth, P. D. Mannion, D. J. Lunt, P. J. Valdes, J. V. Morgan, P. A. Allison, Asteroid impact, not volcanism, caused the end-Cretaceous dinosaur extinction. *Proc. Natl. Acad. Sci. U.S.A.* **117**, 17084–17093 (2020).

28. A. A. Chiarenza, S. L. Brusatte, "Dinosaurs, extinction theories for" in *Reference Module in Life Sciences* (Elsevier, 2023).

29. W. D. Sill, The zoogeography of the Crocodilia. *Copeia* **1968**, 76–88 (1968).

30. L. J. Harmon, J. B. Losos, T. Jonathan Davies, R. G. Gillespie, J. L. Gittleman, W. Bryan Jennings, K. H. Kozak, M. A. McPeek, F. Moreno-Roark, T. J. Near, A. Purvis, R. E. Ricklefs, D. Schluter, J. A. Schulte Ii, O. Seehausen, B. L. Sidlauskas, O. Torres-Carvajal, J. T. Weir, A. Ø. Mooers, Early bursts of body size and shape evolution are rare in comparative data: Early burst of evolution are rare. *Evolution* **64**, 2385–2396 (2010).

31. A. F. C. Howard, M. A. Hurtado-Materon, J. A. Rivera, J. J. Zúñiga-Vega, E. P. Martins, A. M. Lawing, ppgm: An R package for integrating neontological, palaeontological, and climate data in a phylogenetic comparative framework. *Palaeontology* **68**, e70034 (2025).

32. H. Klein, H. Haubold, Archosaur footprints-potential for biochronology of Triassic continental sequences. *Bull. N. M. Mus. Nat. Hist. Sci.* **41**, 120 (2007).

33. H. Haubold, *Ichnia Amphibiorum et Reptiliorum Fossilium* (G. Fischer, 1971). [Tracks of Fossil Amphibians and Reptiles].

34. C. Diedrich, Millions of reptile tracks—Early to Middle Triassic carbonate tidal flat migration bridges of Central Europe—Reptile immigration into the Germanic Basin. *Palaeogeogr. Palaeoclimatol. Palaeoecol.* **259**, 410–423 (2008).

35. M. Avanzini, L. Cavin, A new Isochirotherium trackway from the Triassic of Vieux Emosson, SW Switzerland: Stratigraphic implications. *Swiss J. Geosci.* **102**, 353–361 (2009).

36. F. M. Petti, H. Furrer, E. Collo, E. Martinetto, M. Bernardi, M. Delfino, M. Romano, M. Piazza, Archosauriform footprints in the Lower Triassic of Western Alps and their role in understanding the effects of the Permian-Triassic hyperthermal. *PeerJ* **8**, e10522 (2020).

37. G. Santi, A. Lualdi, A. Decarlis, U. Nicosia, A. Ronchi, Chirotheriid footprints from the Lower-Middle Triassic of the Briançonnais Domain (Pelite di Case Valmarenca, Western Liguria, NW Italy). *Boll. Soc. Paleont. It.* **54**, 82 (2015).

38. E. Mujal, J. Fortuny, A. Bolet, O. Oms, J. Á. López, An archosauromorph dominated ichnoassemblage in fluvial settings from the late Early Triassic of the Catalan Pyrenees (NE Iberian Peninsula). *PLOS ONE* **12**, e0174693 (2017).

39. J. A. Heath, N. Cooper, P. Upchurch, P. D. Mannion, Accounting for sampling heterogeneity suggests a low paleolatitude origin for dinosaurs. *Curr. Biol.* **35**, 941–953.e5 (2025).

40. K. N. Dollman, J. M. Clark, P. A. Viglietti, C. Browning, J. N. Choiniere, Revised anatomy, taxonomy and biostratigraphy of *Notochampsa istedana* Broom, 1904, a Lower Jurassic crocodyliform from the Clarens Formation (Stormberg Group), and its implications for early crocodyliform phylogeny. *J. Syst. Palaeontol.* **19**, 651–675 (2021).

41. C. T. Griffin, B. M. Wynd, D. Munyikwa, T. J. Broderick, M. Zondo, S. Tolan, M. C. Langer, S. J. Nesbitt, H. R. Taruvinga, Africa's oldest dinosaurs reveal early suppression of dinosaur distribution. *Nature* **609**, 313–319 (2022).

42. P. Upchurch, A. A. Chiarenza, A brief review of non-avian dinosaur biogeography: State-of-the-art and prospectus. *Biol. Lett.* **20**, 20240429 (2024).

43. D. Foffa, E. M. Dunne, A. A. Chiarenza, B. M. Wynd, A. Farnsworth, D. J. Lunt, P. J. Valdes, S. J. Nesbitt, B. T. Kligman, A. D. Marsh, W. G. Parker, R. J. Butler, N. C. Fraser, S. L. Brusatte, P. M. Barrett, Climate drivers and palaeobiogeography of lagerpetids and early pterosaurs. *Nat. Ecol. Evol.* **9**, 1359–1372 (2025).

44. M. Bernardi, P. Gianolla, F. M. Petti, P. Mietto, M. J. Benton, Dinosaur diversification linked with the Carnian Pluvial Episode. *Nat. Commun.* **9**, 1499 (2018).

45. M. D. Ezcurra, The phylogenetic relationships of basal archosauromorphs, with an emphasis on the systematics of proterosuchian archosauriforms. *PeerJ* **4**, e1778 (2016).

46. X.-C. Wu, J. Liu, J.-L. Li, The anatomy of the first archosauriform (Diapsida) from the terrestrial Upper Triassic of China. *Vert. PalAs.* **39**, 251–265 (2001).

47. E. H. Colbert, J. T. Gregory, Correlation of continental Triassic sediments by vertebrate fossils. *Geol. Soc. Am. Bull.* **68**, 1456–1467 (1957).

48. W. Parker, P. Clements, "First year results of the ongoing paleontological inventory of Petrified Forest National Park, Arizona" in *The Colorado Plateau: Cultural, Biological, and Physical Research*, C. van Riper, K. L. Cole, Eds. (University of Arizona Press, 2004), pp. 201–210.

49. M. R. Stocker, L.-J. Zhao, S. J. Nesbitt, X.-C. Wu, C. Li, A short-snouted Middle Triassic phytosaur and its implications for the morphological evolution and biogeography of Phytosauria. *Sci. Rep.* **7**, 46028 (2017).

50. S. L. Brusatte, R. J. Butler, T. Sulej, G. Niedźwiedzki, The taxonomy and anatomy of rauisuchian archosaurs from the Late Triassic of Germany and Poland. *Acta Palaeontol. Pol.* **54**, 221–230 (2009).

51. D. Gower, R. Schoch, Postcranial anatomy of the rauisuchian archosaur *Batrachotomus kupferzellensis*. *J. Vertebr. Paleontol.* **29**, 103–122 (2009).

52. M. Antczak, Late Triassic aetosaur (Archosauria) from Krasiejów (SW Poland): New species or an example of individual variation? *Geol. J.* **51**, 779–788 (2016).

53. A. G. Sennikov, On the Pseudosuchians *Tsylmosuchus donensis* and *Scythosuchus basileus* from the Early Triassic of Eastern Europe. *Paleontol. J.* **56**, 91–96 (2022).

54. S. M. Kurzanov, M. B. Efimov, Y. M. Gubin, New archosaurs from the Jurassic of Siberia and Mongolia. *Paleontol. J.* **37**, 53–57 (2003).

55. M. N. Bien, Discovery of Triassic Saurischian and primitive mammalian remains at Lufeng, Yunnan. *Bull. Geol. Soc. China* **20**, 225–234 (1940).

56. Y. Fu, D. Azar, D. Huang, A new species of the extinct family Minlagerrontidae (Insecta: Hemiptera: Cicadomorpha) from mid-Cretaceous Burmese amber. *Cretac. Res.* **107**, 104270 (2020).

57. M. T. Young, J. P. Tennant, S. L. Brusatte, T. J. Challands, N. C. Fraser, N. D. L. Clark, D. A. Ross, The first definitive Middle Jurassic atoposaurid (Crocodylomorpha, Neosuchia), and a discussion on the genus *Theriosuchus*. *Zool. J. Linn. Soc.* **176**, 443–462 (2016).

58. D. Schwarz-Wings, N. Klein, C. Neumann, U. Resch, A new partial skeleton of *Alligatorellus* (Crocodyliformes) associated with echinoids from the Late Jurassic (Tithonian) lithographic limestone of Kelheim, S-Germany. *Foss. Rec.* **14**, 195–205 (2011).

59. J. M. Clark, A new shartegosuchid crocodyliform from the Upper Jurassic Morrison Formation of western Colorado. *Zool. J. Linn. Soc.* **163**, S152–S172 (2011).

60. O. C. Marsh, A new order of extinct reptiles (Macelognatha). *Am. J. Sci.* **s3-27**, 341 (1884).

61. E. C. Drinker, Descriptions of new Vertebrata from the upper Tertiary formations of the West—Biodiversity Heritage Library. *Proc. Am. Philos. Soc.* **17**, 219–231 (1877).

62. K. N. Whetstone, K. N. Whetstone, P. J. Whybrow, A cursorial crocodilian from the Triassic of Lesotho (Basutoland) southern Africa. *Occas. Pap. Mus. Nat. Hist. Univ. Kansas* **106**, 1–37 (1983).

63. A. S. Brink, A new small Thecodont from the Red beds of the Stormberg series. *Palaeontol. Afr.* **6**, 109–115 (1959).

64. S. W. Salisbury, R. E. Molnar, E. Frey, P. M. A. Willis, The origin of modern crocodyliforms: New evidence from the Cretaceous of Australia. *Proc. R. Soc. B* **273**, 2439–2448 (2006).

65. R. Owen, A. S. Woodward, *Note on the Crocodilian Remains Accompanying Dr. T. L. Bell's Paper on Kotah / by Professor Owen* (1852).

66. S. Salisbury, Crocodilians from the Lower Cretaceous (Berriasian) Purbeck Limestone Group of Dorset, southern England. *Spec. Pap. Palaeontol.* **68**, 121–144 (2001).

67. J. E. Martin, M. Delfino, G. Garcia, P. Godefroit, S. Berton, X. Valentin, New specimens of *Allodaposuchus precedens* from France: Intraspecific variability and the diversity of European Late Cretaceous eusuchians. *Zool. J. Linn. Soc.* **176**, 607–631 (2016).

68. S.-L. Sun, S.-W. Chen, Z.-J. Yang, T. Zhang, Y.-F. Li, J.-C. Zhu, H.-C. Wu, T.-T. Wang, Y.-J. Zheng, Q.-H. Ding, Age of the Tuchengzi formation in western Liaoning province and the Jurassic–Cretaceous boundary from the continuous core records of Well YD1, Jinyang Basin. *Minerals* **12**, 953 (2022).

69. L. Xing, M. G. Lockley, T. Du, L. Zhang, H. Klein, A. Romilio, W. S. Persons, K. Wang, Z. Li, X. Wan, Dinosaur tracks from the Jurassic-Cretaceous boundary Tuchengzi Formation (Hebei Province, China) used as building stones in the Chengde imperial summer resort: Age, ichnology, and history. *Cretac. Res.* **107**, 104310 (2020).

70. J. B. van Niekerk, R. T. Tucker, P. Delgerzaya, W. J. Freimuth, T. Chinzorig, K. Tsogtbaatar, J. Yoshida, L. E. Zanno, Geological reassessment of syn-rift extensional sequences in the Shine Usny Tolgod and Dzun Shakhai fossil localities, Eastern Gobi Basin, Magnolia. *Sedimentology* **72**, 1275–1315 (2025).

71. J. P. Tennant, P. D. Mannion, P. Upchurch, Environmental drivers of crocodyliform extinction across the Jurassic/Cretaceous transition. *Proc. R. Soc. B* **283**, 20152840 (2016).

72. B. U. Haq, J. Hardenbol, P. R. Vail, Chronology of fluctuating sea levels since the Triassic. *Science* **235**, 1156–1167 (1987).

73. D. A. Ruban, Mesozoic long-term eustatic cycles and their uncertain hierarchy. *Geosci. Front.* **6**, 503–511 (2015).

74. R. J. Butler, R. B. J. Benson, M. T. Carrano, P. D. Mannion, P. Upchurch, Sea level, dinosaur diversity and sampling biases: Investigating the 'common cause' hypothesis in the terrestrial realm. *Proc. R. Soc. B* **278**, 1165–1170 (2011).

75. H. M. Avrahami, T. A. Gates, A. B. Heckert, P. J. Makovicky, L. E. Zanno, A new microvertebrate assemblage from the Mussentuchit Member, Cedar Mountain Formation: Insights into the paleobiodiversity and paleobiogeography of early Late Cretaceous ecosystems in western North America. *PeerJ* **6**, e5883 (2018).

76. H. C. E. Larsson, H.-D. Sues, Cranial osteology and phylogenetic relationships of *Hamadasuchus rebouli* (Crocodyliformes: Mesoeucrocodylia) from the Cretaceous of Morocco. *Zool. J. Linn. Soc.* **149**, 533–567 (2007).

77. A. W. A. Kellner, A. E. P. Pinheiro, S. A. K. Azevedo, D. D. R. Henriques, L. B. de Carvalho, G. R. Oliveira, A new crocodyliform from the Alcântara Formation (Cenomanian), Cajual Island, Brazil. *Zootaxa* **2030**, 49–58 (2009).

78. N. Ibrahim, P. C. Sereno, D. J. Varricchio, D. M. Martill, D. B. Dutheil, D. M. Unwin, L. Baidder, H. C. E. Larsson, S. Zouhri, A. Kaoukaya, Geology and paleontology of the Upper Cretaceous Kem Kem Group of eastern Morocco. *ZooKeys* **928**, 1–216 (2020).

79. M. A. White, P. R. Bell, N. E. Campione, G. Sansalone, S. A. Birch, J. J. Bevitt, R. E. Molnar, A. G. Cook, S. Wroe, D. A. Elliott, Abdominal contents reveal Cretaceous crocodyliforms ate dinosaurs. *Gondw. Res.* **106**, 281–302 (2022).

80. T. L. Adams, A new neosuchian crocodyliform from the Lower Cretaceous (late Aptian) Twin Mountains Formation of North-Central Texas. *J. Vertebr. Paleontol.* **33**, 85–101 (2013).

81. T. L. Adams, Small crocodyliform from the Lower Cretaceous (Late Aptian) of central Texas and its systematic relationship to the evolution of Eusuchia. *J. Paleo.* **88**, 1031–1049 (2014).

82. R. L. Cifelli, J. D. Gardner, R. L. Nydaml, D. L. Brinkman, Additions to the vertebrate fauna of the Antlers Formation (Lower Cretaceous), southeastern Oklahoma. *Okla. Geol. Notes* **57**, 124–131 (1997).

83. C. A. Brochu, Phylogenetics, taxonomy, and historical biogeography of Alligatoroidea. *J. Vertebr. Paleontol.* **19**, 9–100 (1999).

84. A. P. Cossette, C. A. Brochu, A systematic review of the giant alligatoroid *Deinosuchus* from the Campanian of North America and its implications for the relationships at the root of Crocodylia. *J. Vertebr. Paleontol.* **40**, e1767638 (2020).

85. M. Delfino, J. E. Martin, E. Buffetaut, A new species of Acynodon (Crocodylia) from the upper cretaceous (Santonian–Campanian) of Villaggio del Pescatore, Italy. *Palaeontology* **51**, 1091–1106 (2008).

86. J. E. Martin, New Material of the Late Cretaceous Globidontan *Acynodon iberoccitanus* (Crocodylia) from Southern France. *J. Vertebr. Paleontol.* **27**, 362–372 (2007).

87. M. Muscioni, A. A. Chiarenza, M. Delfino, M. Fabbri, K. Milocco, F. Fanti, *Acynodon adriaticus* from Villaggio del Pescatore (Campanian of Italy): Anatomical and chronostratigraphic integration improves phylogenetic resolution in Hylaeochampsidae (Eusuchia). *Cretac. Res.* **151**, 105631 (2023).

88. A. Ősi, J. M. Clark, D. B. Weishampel, First report on a new basal eusuchian crocodyliform with multicusped teeth from the Upper Cretaceous (Santonian) of Hungary. *N. Jb. Geol. Paläont. Abh.* **243**, 169–177 (2007).

89. M. A. Norell, D. Pol, A. H. Turner, Morphology of the Late Cretaceous crocodylomorph Shamosuchus djadochtaensis and a discussion of neosuchian phylogeny as related to the origin of Eusuchia. *Bull. Am. Mus. Nat. Hist.* **324**, 1–103 (2009).

90. D. Pol, M. Norell, A new gobiosuchid crocodyliform taxon from the Cretaceous of Mongolia. *Am. Mus. Novit.* **3458**, 1–31 (2004).

91. I. de Souza Carvalho, Z. B. de Gasparini, L. Salgado, F. M. de Vasconcellos, T. da Silva Marinho, Climate's role in the distribution of the Cretaceous terrestrial Crocodyliformes throughout Gondwana. *Palaeogeogr. Palaeoclimatol. Palaeoecol.* **297**, 252–262 (2010).

92. D. A. Campos, G. R. Oliveira, R. G. Figueiredo, D. Riff, S. A. K. Azevedo, L. B. Carvalho, A. W. A. Kellner, On a new peirosaurid crocodyliform from the Upper Cretaceous, Bauru Group, southeastern Brazil. *An. Acad. Bras. Ciênc.* **83**, 317–327 (2011).

93. G. A. Buckley, C. A. Brochu, D. W. Krause, D. Pol, A pug-nosed crocodyliform from the Late Cretaceous of Madagascar. *Nature* **405**, 941–944 (2000).

94. G. Buckley, C. Brochu, An enigmatic new crocodile from the Upper Cretaceous of Madagascar. *Cretaceous F. Ver.* **60**, 149–175 (1999).

95. G. V. R. Prasad, F. de Lapparent de Broin, Late Cretaceous crocodile remains from Naskal (India): Comparisons and biogeographic affinities. *Ann. Paléontol.* **88**, 19–71 (2002).

96. G. V. R. Prasad, A. Sharma, O. Verma, A. Khosla, L. R. Singh, R. Priyadarshini, Testudoid and crocodiloid eggshells from the Upper Cretaceous Deccan intertrappean beds of central India. *C. R. Palevol* **14**, 513–526 (2015).

97. A. Khosla, J. J. W. Sertich, G. V. R. Prasad, O. Verma, Dyrosaurid remains from the Intertrappean Beds of India and the Late Cretaceous distribution of Dyrosauridae. *J. Vertebr. Paleontol.* **29**, 1321–1326 (2009).

98. R. V. Hill, J. A. Mccartney, E. Roberts, M. Bouaré, F. Sissoko, M. A. O'leary, Dyrosaurid (Crocodyliformes: Mesoeucrocodylia) fossils from the Upper Cretaceous and Paleogene of Mali: Implications for phylogeny and survivorship across the K/T boundary. *Am. Mus. Novit.* **2008**, 1–19 (2008).

99. S. Jouve, B. Bouya, M. Amaghzaz, A short-snouted dyrosaurid (Crocodyliformes, Mesoeucrocodylia) from the Palaeocene of Morocco. *Palaeontology* **48**, 359–369 (2005).

100. S. Jouve, N. Bardet, N.-E. Jalil, X. P. Suberbiola, B. Bouya, M. Amaghzaz, The oldest African crocodylian: Phylogeny, paleobiogeography, and differential survivorship of marine

reptiles through the Cretaceous-Tertiary boundary. *J. Vertebr. Paleontol.* **28**, 409–421 (2008).

101. R. Otero, S. Soto Acuña, R. Yury, Primer registro de tortugas y cocodrilos En El Eoceno de Magallanes, extremo sur de Chile. *XIII Cong. Geol. Chileno* **3**, (2012). [First record of turtles and crocodiles in the Eocene of Magallanes, southernmost part of Chile].

102. G. G. Simpson, *New Reptiles from the Eocene of South America* (The American Museum of Natural History, 1937).

103. M. B. Efimov, A review of Fossil Crocodiles of Mongolia, *Trudy Sovmestnoi Sovetsko-Mongol'skoi Paleontologicheskoi Ekspeditsii*, (1983), vol. 24, pp. 76–96.

104. M. A. Sallaberry, R. E. Yury-Yáñez, R. A. Otero, S. Soto-Acuña, T. G. Torres, Eocene birds from the western margin of southernmost South America. *J. Paleo.* **84**, 1061–1070 (2010).

105. M. O. Woodburne, W. J. Zinsmeister, Fossil land mammal from Antarctica. *Science* **218**, 284–286 (1982).

106. A. P. Cossette, D. A. Tarailo, Crocodylian diversity during the early Eocene climatic optimum in the Golden Valley Formation of North Dakota, U.S.A. *J. Vertebr. Paleontol.* **44**, e2403579 (2024).

107. C. A. Brochu, Systematics and taxonomy of Eocene tomistomine crocodylians from Britain and northern Europe. *Palaeontology* **50**, 917–928 (2007).

108. J. E. Martin, K. Lauprasert, H. Tong, V. Suteethorn, E. Buffetaut, An Eocene tomistomine from peninsular Thailand. *Ann. Paléontol.* **105**, 245–253 (2019).

109. J. E. Martin, P.-O. Antoine, V. Perrier, J.-L. Welcomme, G. Metais, L. Marivaux, A large crocodyloid from the Oligocene of the Bugti Hills, Pakistan. *J. Vertebr. Paleontol.* **39**, e1671427 (2019).

110. L. Macaluso, J. E. Martin, L. Del Favero, M. Delfino, Revision of the crocodilians from the Oligocene of Monteviale, Italy, and the diversity of European eusuchians across the Eocene-Oligocene boundary. *J. Vertebr. Paleontol.* **39**, e1601098 (2019).

111. T. Massonne, M. Böhme, Re-evaluation of the morphology and phylogeny of *Diplocynodon levantinicum* Huene & Nikoloff, 1963 and the stratigraphic age of the West Maritsa coal field (Upper Thrace Basin, Bulgaria). *PeerJ* **10**, e14167 (2022).

112. C. A. Brochu, P. D. Gingerich, *New Tomistomine Crocodylian from the Middle Eocene (Bartonian) of Wadi Hitan, Fayum Province, Egypt* (University of Michigan, Museum of Paleontology, 2000), vol. 30, pp. 251–268.

113. A. K. Hastings, B. W. Schubert, J. R. Bourque, R. C. Hulbert, Oldest record of *Alligator* in southeastern North America. *Palaeontol. Electron.* **26**, a6 (2023).

114. J. Ristevski, G. J. Price, V. Weisbecker, S. W. Salisbury, First record of a tomistomine crocodylian from Australia. *Sci. Rep.* **11**, 12158 (2021).

115. P. D. Mannion, A. A. Chiarenza, P. L. Godoy, Y. N. Cheah, Spatiotemporal sampling patterns in the 230 million year fossil record of terrestrial crocodylomorphs and their impact on diversity. *Palaeontology* **62**, 615–637 (2019).

116. T. Mörs, F. Hocht, B. Wutzler, Die erste Wirbeltierfauna aus der miozänen Braunkohle der Niederrheinischen Bucht (Ville-Schichten, Tagebau Hambach). *Paläontol. Z.* **74**, 145–170 (2000). [The first vertebrate fauna from the Miocene lignite of the Lower Rhine Embayment (Ville strata, Hambach open-cast mine)].

117. M. Böhme, The Miocene climatic optimum: Evidence from ectothermic vertebrates of Central Europe. *Palaeogeogr. Palaeoclimatol. Palaeoecol.* **195**, 389–401 (2003).

118. G. Lio, A. Federico, C. N. Roberto, C. P. Anselmo, The youngest crocodyliform record from Patagonia (Chichinales Formation, Early Miocene). *Braz. Geogr. J.* **8**, 136–143 (2019).

119. T. H. Worthy, R. Paul Scofield, V. L. De Pietri, S. W. Salisbury, W. Schwarzhans, S. J. Hand, M. Archer, A synopsis of the Early Miocene St Bathans Fauna of New Zealand. *Geobios* **90**, 163–178 (2025).

120. R. E. Molnar, M. Pole, A Miocene crocodilian from New Zealand. *Alcheringa* **21**, 65–70 (1997).

121. S. J. Godfrey, *The Geology and Vertebrate Paleontology of Calvert Cliffs, Maryland, USA* (Smithsonian Institution Scholarly Press, 2018).

122. T. Nakagawa, Y. Sowa, Crocodilian tooth from the Miocene Uchiura Group, in the western part of Fukui Prefecture, Central Japan. *Mizunami Fossil Mus.* **37**, 189–191 (2011).

123. M. H. L. Pickford, B. Senut, P. Mein, D. Gommery, J. Morales, D. Soria, M. Nieto-Diaz, J. D. Ward, M. H. L. Pickford, B. Senut, P. Mein, D. Gommery, J. Morales, D. Soria, M. Nieto-Diaz, J. D. Ward, Preliminary results of new excavations at Arrisdrift, middle Miocene of southern Namibia. *C. R. Acad. Sci. Ser. II Sci. Terre Planetes* **322**, 991–996 (1996).

124. M. Pickford, A new species of crocodile from Early and Middle Miocene deposits of the Lower Orange River Valley, Namibia, and the origins of the Nile crocodile (*Crocodylus niloticus*). *Mem. Geol. Surv. Namib.* **19**, 51–65 (2003).

125. M. Harzhauser, U. B. Göhlich, M. Gross, D. Vasilyan, The last crocodylian in Central Europe? A new occurrence from the late Middle Miocene of the Vienna Basin (Austria). *Hist. Biol.* **37**, 2751–2758 (2025).

126. M. Górka, J. Březina, M. Chroust, R. Kowalski, S. López-Torres, M. Tałanda, Crocodylian remains from the Miocene of the Fore-Carpathian Basin and its foreland—Including the world's northernmost Neogene crocodylian. *Acta Palaeontol. Pol.* **70**, 225–251 (2025).

127. G. S. Morgan, R. B. Ridgway, *Late Pliocene* (*Late Blancan*) *Vertebrates from the St. Petersburg Times Site*, *Pinellas County*, *Florida*, *with a Brief Review of Florida Blancan Faunas* (Florida Paleontological Society, 1987).

128. F. Barrios, Presencia de *Caiman latirostris* (Daudin, 1802) (Crocodylia, Alligatoridae) en la Formación Piquete (Plioceno-Pleistoceno Temprano) de la Provincia De Salta, Argentina: Implicancias Paleoambientales y Sistemáticas. *Ameghiniana* **50**, 522–534 (2013). [Presence of Caiman latirostris (Daudin, 1802) (Crocodylia, Alligatoridae) in the Piquete Formation (Pliocene-Early Pleistocene) of the Province of Salta, Argentina: Paleoenvironmental and Systematic Implications].

129. T. G. Bromage, F. Schrenk, Y. M. Juwayeyi, Paleobiogeography of the Malawi Rift: Age and vertebrate paleontology of the Chiwondo Beds, northern Malawi. *J. Hum. Evol.* **28**, 37–57 (1995).

130. R. Srivastava, H. Schleich, *Fossil Turtles and Crocodiles from the Caenozoic of S-Asia* (ARCO-Nepal, 2018).

131. M. Iijima, A. Momohara, Y. Kobayashi, S. Hayashi, T. Ikeda, H. Taruno, K. Watanabe, M. Tanimoto, S. Furui, Toyotamaphimeia cf. machikanensis (Crocodylia, Tomistominae) from the Middle Pleistocene of Osaka, Japan, and crocodylian survivorship through the Pliocene-Pleistocene climatic oscillations. *Palaeogeogr. Palaeoclimatol. Palaeoecol.* **496**, 346–360 (2018).

132. C. K. Brain, *The Hunters or the Hunted?: An Introduction to African Cave Taphonomy* (University of Chicago Press, 1983).

133. J. W. Gidley, *Preliminary Report on a Recently Discovered Pleistocene Cave Deposit near Cumberland, Maryland* (US Government Printing Office, 1913).

134. J. Balouet, E. Buffetaut, *Mekosuchus inexpectatus*, n. Gen., n. Sp., crocodilien nouveau de l'Holocene de Nouvelle Calédonie. *C. R. Acad. Sci. Paris Ser. 2* **304**, 853–856 (1987). [Mekosuchus inexpectatus, n. Gen., n. Sp., a new crocodilian from the Holocene of New Caledonia].

135. P. Vinca, The Stone Age of Mount Carmel: Excavations at the Wady Elmughara, Vol. I, by D. A. E. Garrod and D. M. A. Bate. pp. 240, pl. LXV, text figs. 8. Clarendon Press, Oxford, 1937. Price, 42s. *Proc. Prehist. Soc.* (1937), vol. 3, pp. 486–488.

136. T. F. Hansen, Stabilizing selection and the comparative analysis of adaptation. *Evolution* **51**, 1341–1351 (1997).

137. N. Cooper, R. P. Freckleton, W. Jetz, Phylogenetic conservatism of environmental niches in mammals. *Proc. R. Soc. B* **278**, 2384–2391 (2011).

138. H. C. Jenkyns, Geochemistry of oceanic anoxic events. *Geochem. Geophys. Geosyst.* **11**, Q03004 (2010).

139. J. C. Zachos, G. R. Dickens, R. E. Zeebe, An early Cenozoic perspective on greenhouse warming and carbon-cycle dynamics. *Nature* **451**, 279–283 (2008).

140. J. Sun, W. Liu, Z. Guo, L. Qi, Z. Zhang, Enhanced aridification across the Eocene/Oligocene transition evidenced by geochemical record in the Tajik Basin, Central Asia. *Glob. Planet. Change* **211**, 103789 (2022).

141. J. Ristevski, P. M. A. Willis, A. M. Yates, M. A. White, L. J. Hart, M. D. Stein, G. J. Price, S. W. Salisbury, Migrations, diversifications and extinctions: The evolutionary history of crocodyliforms in Australasia. *Alcheringa* **47**, 370–415 (2023).

142. H. A. Martin, Cenozoic climatic change and the development of the arid vegetation in Australia. *J. Arid Environ.* **66**, 533–563 (2006).

143. A. T. Cribb, K. K. Formoso, C. H. Woolley, J. Beech, S. Brophy, P. Byrne, V. C. Cassady, A. L. Godbold, E. Larina, P. Maxeiner, Y.-H. Wu, F. A. Corsetti, D. J. Bottjer, Contrasting terrestrial and marine ecospace dynamics after the end-Triassic mass extinction event. *Proc. R. Soc. B* **290**, 20232232 (2023).

144. J. H. F. L. Davies, A. Marzoli, H. Bertrand, N. Youbi, M. Ernesto, U. Schaltegger, End-Triassic mass extinction started by intrusive CAMP activity. *Nat. Commun.* **8**, 15596 (2017).

145. M. Hautmann, "Extinction: End-Triassic mass extinction" in *Encyclopedia of Life Sciences* (John Wiley & Sons, Ltd., 2012).

146. S. J. Nesbitt, *The Early Evolution of Archosaurs: Relationships and the Origin of Major Clades* (American Museum of Natural History, 2011).

147. S. D. Schoepfer, T. J. Algeo, B. van de Schootbrugge, J. H. Whiteside, The Triassic–Jurassic transition—A review of environmental change at the dawn of modern life. *Earth Sci. Rev.* **232**, 104099 (2022).

148. S. L. Brusatte, M. J. Benton, M. Ruta, G. T. Lloyd, The first 50 Myr of dinosaur evolution: Macroevolutionary pattern and morphological disparity. *Biol. Lett.* **4**, 733–736 (2008).

149. S. L. Brusatte, M. J. Benton, G. T. Lloyd, M. Ruta, S. C. Wang, Macroevolutionary patterns in the evolutionary radiation of archosaurs (Tetrapoda: Diapsida). *Earth Environ. Sci. Trans. R. Soc. Edinb.* **101**, 367–382 (2010).

150. L. Budic, C. F. Dormann, Climatic niches in phylogenetic comparative studies: A review of challenges and approaches. bioRxiv 018796 [Preprint] (2015). https://doi.org/10.1101/018796.

151. J. J. Wiens, Speciation and ecology revisited: Phylogenetic niche conservatism and the origin of species. *Evolution* **58**, 193–197 (2004).

152. M. D. Crisp, L. G. Cook, Phylogenetic niche conservatism: What are the underlying evolutionary and ecological causes? *New Phytol.* **196**, 681–694 (2012).

153. E. A. Hadly, P. A. Spaeth, C. Li, Niche conservatism above the species level. *Proc. Natl. Acad. Sci. U.S.A.* **106**, 19707–19714 (2009).

154. A. B. Smith, W. Godsoe, F. Rodríguez-Sánchez, H.-H. Wang, D. Warren, Niche estimation above and below the species level. *Trends Ecol. Evol.* **34**, 260–273 (2019).

155. A. T. Peterson, J. Soberón, R. G. Pearson, R. P. Anderson, E. Martínez-Meyer, M. Nakamura, M. B. Araújo, *Ecological Niches and Geographic Distributions* (*MPB-49*) (Princeton Univ. Press, 2011).

156. N. Sillero, What does ecological modelling model? A proposed classification of ecological niche models based on their underlying methods. *Ecol. Model.* **222**, 1343–1346 (2011).

157. K. Rodiouchkina, S. Goderis, C. B. Senel, P. Kaskes, Ö. Karatekin, M. E. Böttcher, I. Rodushkin, J. Vellekoop, P. Claeys, F. Vanhaecke, Reduced contribution of sulfur to the mass extinction associated with the Chicxulub impact event. *Nat. Commun.* **16**, 620 (2025).

158. L. W. Alvarez, W. Alvarez, F. Asaro, H. V. Michel, Extraterrestrial cause for the Cretaceous-Tertiary extinction. *Science* **208**, 1095–1108 (1980).

159. A. A. Chiarenza, S. L. Brusatte, "Dinosaurs, extinction theories for" in *Encyclopedia of Biodiversity* (*Third Edition*), S. M. Scheiner, Ed. (Academic Press, 2024), pp. 298–309.

160. S. Saber, B. S. Salem, K. Ouda, A. S. Gohar, S. El-Sayed, P. M. O'Connor, H. M. Sallam, An early dyrosaurid (*Wadisuchus kassabi* gen. et sp. nov.) from the Campanian of Egypt sheds light on the origin and biogeography of Dyrosauridae. *Zool. J. Linn. Soc.* **205**, zlaf134 (2025).

161. G. G. Bravo, D. Pol, J. M. Leardi, J. M. Krause, C. S. C. Nicholl, G. Rougier, P. D. Mannion, A new notosuchian crocodyliform from the Early Palaeocene of Patagonia and the survival of a large-bodied terrestrial lineage across the K–Pg mass extinction. *Proc. R. Soc. B* **292**, 20241980 (2025).

162. E. E. Saupe, V. Barve, C. E. Myers, J. Soberón, N. Barve, C. M. Hensz, A. T. Peterson, H. L. Owens, A. Lira-Noriega, Variation in niche and distribution model performance: The need for a priori assessment of key causal factors. *Ecol. Model.* **237-238**, 11–22 (2012).

163. A. M. Lawing, P. D. Polly, D. K. Hews, E. P. Martins, Including fossils in phylogenetic climate reconstructions: A deep time perspective on the climatic niche evolution and diversification of spiny lizards (Sceloporus). *Am. Nat.* **188**, 133–148 (2016).

164. J. Elith, M. Kearney, S. Phillips, The art of modelling range-shifting species. *Methods Ecol. Evol.* **1**, 330–342 (2010).

165. N. Sillero, J. Ribeiro-Silva, S. Arenas-Castro, Shifts in climatic realised niches of Iberian species. *Oikos* **2022**, e08505 (2022).

166. N. Sillero, S. Arenas-Castro, U. Enriquez-Urzelai, C. G. Vale, D. Sousa-Guedes, F. Martínez-Freiría, R. Real, A. M. Barbosa, Want to model a species niche? A step-by-step guideline on correlative ecological niche modelling. *Ecol. Model.* **456**, 109671 (2021).

167. N. Sillero, R. dos Santos, A. C. Teodoro, M. A. Carretero, Ecological niche models improve home range estimations. *J. Zool.* **313**, 145–157 (2021).

168. S. Maidment, R. J. Butler, New frontiers in dinosaur exploration. *Biol. Lett.* **21**, 20250045 (2025).

169. S. Varela, J. M. Lobo, J. Hortal, Using species distribution models in paleobiogeography: A matter of data, predictors and concepts. *Palaeogeogr. Palaeoclimatol. Palaeoecol.* **310**, 451–463 (2011).

170. S. Chamberlain , D. Oldoni , V. Barve , P. Desmet, L. Geffert, D. Mcglinn, K. Ram, rOpenSci, J. Waller, rgbif: Interface to the global biodiversity information facility API, version 3.8.3 (2025). https://cran.r-project.org/web/packages/rgbif/index.html.

171. L. A. Jones, C. D. Dean, B. J. Allen, H. B. Drage, J. T. Flannery-Sutherland, W. Gearty, A. A. Chiarenza, E. M. Dillon, B. M. Farina, P. L. Godoy, Ten simple rules to follow when cleaning occurrence data in palaeobiology. *Palaeontology* **68**, e70028 (2025).

172. A. A. Chiarenza, P. D. Mannion, D. J. Lunt, A. Farnsworth, L. A. Jones, S.-J. Kelland, P. A. Allison, Ecological niche modelling does not support climatically-driven dinosaur diversity decline before the Cretaceous/Paleogene mass extinction. *Nat. Commun.* **10**, 1091 (2019).

173. A. A. Chiarenza, P. D. Mannion, A. Farnsworth, M. T. Carrano, S. Varela, Climatic constraints on the biogeographic history of Mesozoic dinosaurs. *Curr. Biol.* **32**, 570–585.e3 (2022).

174. GBIF backbone taxonomy. doi:10.15468/39omei.

175. A. R. D. Payne, P. D. Mannion, G. T. Lloyd, K. E. Davis, Decoupling speciation and extinction reveals both abiotic and biotic drivers shaped 250 million years of diversity in crocodile-line archosaurs. *Nat. Ecol. Evol.* **8**, 121–132 (2024).

176. C. Scotese, N. Wright, PaleoDEM Resource—Scotese and Wright (2018)—EarthByte (2018). https://www.earthbyte.org/paleodem-resource-scotese-and-wright-2018/.

177. C. Scotese, *PALEOMAP PaleoAtlas for GPlates and the PaleoData Plotter Program* (Geological Society of America, 2016).

178. L. A. Jones, W. Gearty, B. J. Allen, K. Eichenseer, C. D. Dean, S. Galván, M. Kouvari, P. L. Godoy, C. S. C. Nicholl, L. Buffan, E. M. Dillon, J. T. Flannery-Sutherland, A. A., Chiarenza, palaeoverse: A community-driven R package to support palaeobiological analysis. *Methods Ecol. Evol.* **14**, 2205–2215 (2023).

179. A. Chiarenza, "Virtual habitats, fossil preservation, and estimates of dinosaur biodiversity in the Cretaceous of North America," thesis, Imperial College London (2019).

180. M. D. Cox, A primitive equation, 3-dimensional model of the ocean (GFDL Ocean Group Technical Report No. 1, Geophysical Fluid Dynamics Laboratory, 1984).

181. D. O. Gough, Solar interior structure and luminosity variations. *Solar Phys.* **74**, 21–34 (1981).

182. J. W. B. Rae, Y. G. Zhang, X. Liu, G. L. Foster, H. M. Stoll, R. D. M. Whiteford, Atmospheric CO_2 over the past 66 million years from marine archives. *Annu. Rev. Earth Planet. Sci.* **49**, 609–641 (2021).

183. G. L. Foster, D. L. Royer, D. J. Lunt, Future climate forcing potentially without precedent in the last 420 million years. *Nat. Commun.* **8**, 14845 (2017).

184. A. K. Smith, D. R. Marsh, J. M. Russell III, M. G. Mlynczak, F. J. Martin-Torres, E. Kyrölä, Satellite observations of high nighttime ozone at the equatorial mesopause. *J. Geophys. Res. Atmos.* **113**, (2008).

185. N. Sagoo, P. Valdes, R. Flecker, L. J. Gregoire, The Early Eocene equable climate problem: Can perturbations of climate model parameters identify possible solutions? *Phil. Trans. R. Soc. A* **371**, 20130123 (2013).

186. J. T. Kiehl, C. A. Shields, Sensitivity of the Palaeocene–Eocene Thermal Maximum climate to cloud properties. *Phil. Trans. R. Soc. A* **371**, 20130093 (2013).

187. A. Farnsworth, D. J. Lunt, C. L. O'Brien, G. L. Foster, G. N. Inglis, P. Markwick, R. D. Pancost, S. A. Robinson, Climate sensitivity on geological timescales controlled by nonlinear feedbacks and ocean circulation. *Geophys. Res. Lett.* **46**, 9880–9889 (2019).

188. P. J. Valdes, C. R. Scotese, D. J. Lunt, Deep ocean temperatures through time. *Clim. Past* **17**, 1483–1506 (2021).

189. R. J. Hijmans, M. Barbosa, R. Bivand, A. Brown, M. Chirico, E. Cordano, K. Dyba, E. Pebesma, B. Rowlingson, M. D. Sumner, terra: Spatial data analysis, version 1.8-86 (2025). https://cran.r-project.org/web/packages/terra/index.html.

190. A. T. Kocsis, N. B. Raja, S. Williams, E. M. Dowding, D. Forschungsgemeinschaft, F. G. Nordbayern, rgplates: R interface for the GPlates web service and desktop application, version 0.6.1 (2025). https://cran.r-project.org/web/packages/rgplates/index.html.

191. C. R. Johnson, Thermoregulation in crocodilians—I. Head-body temperature control in the papuan-new guinean crocodiles, *Crocodylus novaeguineae* and *Crocodylus porosus*. *Comp. Biochem. Physiol. A Physiol.* **49**, 3–28 (1974).

192. E. N. Smith, Behavioral and physiological thermoregulation of crocodilians. *Am. Zool.* **19**, 239–247 (1979).

193. K. E. Barham, R. G. Dwyer, C. H. Frere, L. K. Bentley, C. J. Baker, H. A. Campbell, T. R. Irwin, C. E. Franklin, Cooling down is as important as warming up for a large-bodied tropical reptile. *Proc. R. Soc. B* **291**, 20241804 (2024).

194. K. E. Barham, C. H. Frère, R. G. Dwyer, C. J. Baker, H. A. Campbell, T. R. Irwin, C. E. Franklin, Climate-induced shifts in crocodile body temperature impact behavior and performance. *Curr. Biol.* **35**, 1164–1170.e2 (2025).

195. D. A. Kring, The Chicxulub impact event and its environmental consequences at the Cretaceous–Tertiary boundary. *Palaeogeogr. Palaeoclimatol. Palaeoecol.* **255**, 4–21 (2007).

196. T. J. Goldin, H. J. Melosh, Self-shielding of thermal radiation by Chicxulub impact ejecta: Firestorm or fizzle? *Geology* **37**, 1135–1138 (2009).

197. W. Thuiller, D. Georges, M. Gueguen, R. Engler, F. Breiner, biomod2: Ensemble platform for species distribution modeling, version 3.5.1 (2021). https://CRAN.R-project.org/package=biomod2.

198. K. H. Kozak, J. J. Wiens, Accelerated rates of climatic-niche evolution underlie rapid species diversification. *Ecol. Lett.* **13**, 1378–1389 (2010).

199. R. Dawkins, *The Extended Phenotype: The Long Reach of the Gene* (Oxford Univ. Press, 2016).

200. A. L. S. Meyer, M. R. Pie, Climate change estimates surpass rates of climatic niche evolution in primates. *Int. J. Primatol.* **43**, 40–56 (2022).

201. M. R. Pie, L. L. F. Campos, A. L. S. Meyer, A. Duran, The evolution of climatic niches in squamate reptiles. *Proc. R. Soc. B* **284**, 20170268 (2017).

202. A. A. Chiarenza, J. L. Cantalapiedra, L. A. Jones, S. Gamboa, S. Galván, A. J. Farnsworth, P. J. Valdes, G. Sotelo, S. Varela, Early Jurassic origin of avian endothermy and thermophysiological diversity in dinosaurs. *Curr. Biol.* **34**, 2517–2527.e4 (2024).

203. G. E. Hutchinson, Homage to Santa Rosalia or why are there so many kinds of animals? *Am. Nat.* **93**, 145–159 (1959).

204. G. E. Hutchinson, Concluding remarks. *Cold Spring Harb. Symp. Quant. Biol.* **22**, 415–427 (1957).

205. A. M. Lawing, A. Howard, M.-A. Hurtado-Materon, ppgm: PaleoPhyloGeographic modeling of climate niches and species distributions, version 1.1 (2025). https://cran.r-project.org/web/packages/ppgm/index.html.

206. S. P. Blomberg, T. Garland Jr., A. R. Ives, Testing for phylogenetic signal in comparative data: Behavioral traits are more labile. *Evolution* **57**, 717–745 (2003).

207. L. J. Revell, phytools: An R package for phylogenetic comparative biology (and other things). *Methods Ecol. Evol.* **3**, 217–223 (2012).

208. L. J. Harmon, J. T. Weir, C. D. Brock, R. E. Glor, W. Challenger, GEIGER: Investigating evolutionary radiations. *Bioinformatics* **24**, 129–131 (2008).

209. J. C. Uyeda, L. J. Harmon, A novel Bayesian method for inferring and interpreting the dynamics of adaptive landscapes from phylogenetic comparative data. *Syst. Biol.* **63**, 902–918 (2014).

210. T. Ingram, D. L. Mahler, SURFACE: Detecting convergent evolution from comparative data by fitting Ornstein-Uhlenbeck models with stepwise Akaike Information Criterion. *Methods Ecol. Evol.* **4**, 416–425 (2013).

211. J. M. Beaulieu, B. O'Meara, OUwie: Analysis of evolutionary rates in an OU framework, version 2.10 (2022). https://cran.r-project.org/web/packages/OUwie/index.html.

212. J. Beaulieu, thej022214/OUwie (2026). https://github.com/thej022214/OUwie.

213. A. Forster, S. Schouten, M. Baas, K. Moriya, P. A. Wilson, J. S. Sinninghe Damsté, The cretaceous thermal maximum and oceanic anoxic event 2 in the tropics: Sea-surface temperature and stable organic carbon isotopic records from the equatorial Atlantic. *AGU Fall Meet. Abstr.* (2006).

214. P. Schulte, L. Alegret, I. Arenillas, J. A. Arz, P. J. Barton, P. R. Bown, T. J. Bralower, G. L. Christeson, P. Claeys, C. S. Cockell, G. S. Collins, A. Deutsch, T. J. Goldin, K. Goto, J. M. Grajales-Nishimura, R. A. F. Grieve, S. P. S. Gulick, K. R. Johnson, W. Kiessling, C. Koeberl, D. A. Kring, K. G. MacLeod, T. Matsui, J. Melosh, A. Montanari, J. V. Morgan, C.

R. Neal, D. J. Nichols, R. D. Norris, E. Pierazzo, G. Ravizza, M. Rebolledo-Vieyra, W. U. Reimold, E. Robin, T. Salge, R. P. Speijer, A. R. Sweet, J. Urrutia-Fucugauchi, V. Vajda, M. T. Whalen, P. S. Willumsen, The Chicxulub asteroid impact and mass extinction at the Cretaceous-Paleogene boundary. *Science* **327**, 1214–1218 (2010).

215. F. A. McInerney, S. L. Wing, The Paleocene-Eocene thermal maximum: A perturbation of carbon cycle, climate, and biosphere with implications for the future. *Annu. Rev. Earth Planet. Sci.* **39**, 489–516 (2011).

216. D. R. Prothero, The late eocene-oligocene extinctions. *Annu. Rev. Earth Planet. Sci.* **22**, 145–165 (1994).

217. F. Scheiner, M. Havelcová, K. Holcová, N. Doláková, S. Nehyba, L. Ackerman, J. Trubač, Š. Hladilová, J. Rejšek, T. Utescher, Evolution of palaeoclimate, palaeoenvironment and vegetation in Central Europe during the Miocene Climate Optimum. *Palaeogeogr. Palaeoclimatol. Palaeoecol.* **611**, 111364 (2023).

218. C. R. Scotese, C. Vérard, L. Burgener, R. P. Elling, A. T. Kocsis, The Cretaceous world: Plate tectonics, palaeogeography and palaeoclimate. *Geol. Soc. Lond. Spec. Publ.* **544**, 31–202 (2025).

219. W. Gearty, L. A. Jones, rphylopic: An R package for fetching, transforming, and visualising PhyloPic silhouettes. *Methods Ecol. Evol.* **14**, 2700–2708 (2023).

220. M. B. Lacerda, C. L. Schultz, C. Bertoni-Machado, First "Rauisuchian" archosaur (Pseudosuchia, Loricata) for the Middle Triassic *Santacruzodon* Assemblage Zone (Santa Maria Supersequence), Rio Grande do Sul State, Brazil. *PLOS ONE* **10**, e0118563 (2015).

221. A. C. Mancuso, R. B. Irmis, T. E. Pedernera, L. C. Gaetano, C. A. Benavente, B. T. Breeden III, Paleoenvironmental and biotic changes in the Late Triassic of Argentina: Testing hypotheses of abiotic forcing at the basin scale. *Front. Earth Sci.* **10**, e883788 (2022).

222. R. T. Müller, A new small-sized predatory pseudosuchian archosaur from the Middle-Late Triassic of Southern Brazil. *Sci. Rep.* **14**, 12706 (2024).

223. I. Kuzmin, P. Skutschas, O. I. Grigorieva, S. Krasnolutskii, Goniopholidid crocodylomorph from the Middle Jurassic Berezovsk Quarry locality (Western Siberia, Russia). *Proc. Zool. Inst. RAS* **317**, 452–458 (2013).

224. E. Buffetaut, M. Martin, O. Monod, Phytosaur remains from the Cenger Formation of the Lycian Taurus (western Turkey): Stratigraphical implications. *Geobios* **21**, 237–243 (1988).

225. O. Alcober, Redescription of the skull of *Saurosuchus galilei* (Archosauria: Rauisuchidae). *J. Vertebr. Paleontol.* **20**, 302–316 (2000).

226. A. Lecuona, M. D. Ezcurra, R. B. Irmis, Revision of the early crocodylomorph *Trialestes* romeri (Archosauria, Suchia) from the lower Upper Triassic Ischigualasto Formation of Argentina: One of the oldest-known crocodylomorphs. *Pap. Palaeontol.* **2**, 585–622 (2016).

227. D. A. Ponce, T. M. Scheyer, I. A. Cerda, J. B. Desojo, Palaeobiological inferences of "rauisuchians" *Fasolasuchus tenax* (Los Colorados Fm., Argentina) and *Prestosuchus chiniquensis* (Santa Maria Super sequence, Brazil) from the Middle–Upper Triassic of South America based on microstructural analyses. *J. Anat.* **243**, 893–909 (2023).

228. V. D. Paes Neto, J. B. Desojo, A. C. B. Brust, A. M. Ribeiro, C. L. Schultz, M. B. Soares, The first braincase of the basal aetosaur *Aetosauroides scagliai* (Archosauria: Pseudosuchia) from the Upper Triassic of Brazil. *J. Vertebr. Paleontol.* **41**, e1928681 (2021).

229. J. M. Leardi, I. Yáñez, D. Pol, South American Crocodylomorphs (Archosauria; Crocodylomorpha): A review of the early fossil record in the continent and its relevance on understanding the origins of the clade. *J. South Am. Earth Sci.* **104**, 102780 (2020).

230. F. Tolchard, S. J. Nesbitt, J. B. Desojo, P. Viglietti, R. J. Butler, J. N. Choiniere, 'Rauisuchian' material from the lower Elliot Formation of South Africa and Lesotho: Implications for Late Triassic biogeography and biostratigraphy. *J. Afr. Earth Sci.* **160**, 103610 (2019).

231. K. C. Burmeister, J. J. Flynn, J. M. Parrish, A. R. Wyss, Paleogeographic and biostratigraphic implications of new Early Mesozoic vertebrates from Poamay, central Morondava Basin, Madagascar. *N. M. Mus. Nat. Hist. Sci. Bull.* **37**, 457–475 (2006).

232. A. Sharma, S. Singh, S. S. R., The first turiasaurian sauropod of India reported from the Middle Jurassic (Bathonian) sediments of Jaisalmer Basin, Rajasthan, India. *N. Jb. Geol. Paläontol. Abh.* , 187–203 (2022).

233. S. Maganuco, C. Dal Sasso, A new large predatory archosaur from the Middle Jurassic (Bathonian) of Madagascar. *Atti Soc. It. Sci. Nat. Mus. Civ. St. Nat. Milano* **147**, 19–51 (2006).

234. P. C. Sereno, H. C. E. Larsson, Cretaceous crocodyliforms from the Sahara. *ZooKeys* **28**, 1–143 (2009).

235. B. U. Haq, Cretaceous eustasy revisited. *Global Planet. Change* **113**, 44–58 (2014).

236. R. T. Tucker, J. L. Crowley, M. T. Mohr, R. K. Renaut, P. J. Makovicky, L. E. Zanno, Exceptional age constraint on a fossiliferous sedimentary succession preceding the Cretaceous Thermal Maximum. *Geology* **51**, 962–967 (2023).

237. E. Saura, D. Garcia-Castellanos, E. Casciello, V. Parravano, A. Urruela, J. Vergés, Modeling the flexural evolution of the Amiran and Mesopotamian foreland basins of NW Zagros (Iran-Iraq). *Tectonics* **34**, 377–395 (2015).

238. A. Delgado Buscalioni, P. Piras, R. Vullo, M. Signore, C. Barbera, Early Eusuchia Crocodylomorpha from the vertebrate-rich Plattenkalk of Pietraroia (Lower Albian, southern Appenines, Italy). *Zool. J. Linn. Soc.* **163**, 199–227 (2011).

239. R. Amiot, E. Buffetaut, C. Lécuyer, X. Wang, L. Boudad, Z. Ding, F. Fourel, S. Hutt, F. Martineau, M. A. Medeiros, J. Mo, L. Simon, V. Suteethorn, S. Sweetman, H. Tong, F. Zhang, Z. Zhou, Oxygen isotope evidence for semi-aquatic habits among spinosaurid theropods. *Geology* **38**, 139–142 (2010).

240. M. Rabi, N. Sebők, A revised Eurogondwana model: Late Cretaceous notosuchian crocodyliforms and other vertebrate taxa suggest the retention of episodic faunal links between Europe and Gondwana during most of the Cretaceous. *Gondw. Res.* **28**, 1197–1211 (2015).

241. M. Muscioni, A. A. Chiarenza, C. S. C. Nicholl, T. Perentin, D. Dreossi, F. Fanti, A ziphodont crocodylomorph from Villaggio del Pescatore Lagerstätte (Campanian, Italy). *Zool. J. Linn. Soc.* **205**, zlaf171 (2025).

242. M. V. de Araújo Sena, R. C. L. P. De Andrade, L. B. De Carvalho, S. A. K. De Azevedo, J. M. Sayão, U. G. R. Oliveira, Paleohistology of the crocodyliform *Mariliasuchus amarali* Carvalho & Bertini, 1999 (Mesoeucrocodylia, Notosuchia) based on a new specimen from the Upper Cretaceous of Brazil. *Comptes Rendus. Palevol.* **21**, 349–361 (2022).

243. I. de Souza Carvalho, A. C. A. Campos, P. H. Nobre, Baurusuchus salgadoensis, a new crocodylomorpha from the Bauru Basin (Cretaceous), Brazil. *Gondwana Res.* **8**, 11–30 (2005).

244. F. E. Novas, D. Pol, F. L. Agnolín, I. de Souza Carvalho, M. Manabe, T. Tsuihiji, S. Rozadilla, G. L. Lio, M. P. Isasi, A new large hypercarnivorous crocodyliform from the Maastrichtian of Southern Patagonia, Argentina. *PLOS ONE* **20**, e0328561 (2025).

245. R. T. McCrea, S. G. Pemberton, P. J. Currie, New Ichnotaxa of Mammal and Reptile Tracks from the Upper Paleocene of Alberta. *Ichnos* **11**, 323–339 (2004).

246. C. A. Brochu, A review of "Leidyosuchus" (Crocodyliformes, Eusuchia) from the Cretaceous through Eocene of North America. *J. Vertebr. Paleontol.* **17**, 679–697 (1997).

247. Y. Wang, C. Sullivan, J. Liu, Taxonomic revision of *Eoalligator* (Crocodylia, Brevirostres) and the paleogeographic origins of the Chinese alligatoroids. *PeerJ* **4**, e2356 (2016).

248. J. E. Martin, T. Smith, F. de Lapparent, F. de Broin, M. D. Escuillié, Late Palaeocene eusuchian remains from Mont de Berru, France, and the origin of the alligatoroid Diplocynodon. *Zool. J. Linn. Soc.* **172**, 867–891 (2014).

249. M. Delfino, J. E. Martin, F. de Lapparent de Broin, T. Smith, Evidence for a pre-PETM dispersal of the earliest European crocodyloids. *Hist. Biol.* **31**, 845–852 (2019).

250. R. Estes, J. Howard Hutchison, Eocene lower vertebrates from Ellesmere Island, Canadian Arctic Archipelago. *Palaeogeogr. Palaeoclimatol. Palaeoecol.* **30**, 325–347 (1980).

251. S. L. Walsh, "Middle Eocene mammalian faunas of San Diego County, California" in *The Terrestrial Eocene-Oligocene Transition in North America*, D. R. Prothero, R. J. Emry, Eds. (Cambridge Univ. Press, ed. 1, 1996), pp. 75–119.

252. G. G. Simpson, A new crocodilian from the Notostylops beds of Patagonia. *Publ. Scarritt Exped.* (1933).

253. P. R. Bjork, Latest Eocene vertebrates from northwestern South Dakota. *J. Paleo.* **41**, 227–235 (1967).

254. J. A. Holman, Herpetofauna of the Wood Mountain Formation (Upper Miocene) of Saskatchewan. *Can. J. Earth Sci.* **7**, 1317–1325 (1970).

255. Details—Description of two squalodonts recently discovered in the Calvert Cliffs, Maryland; and notes on the shark-toothed cetaceans—Biodiversity Heritage Library. https://www.biodiversitylibrary.org/part/53955.

256. A. Forasiepi, A. Martinelli, M. de la Fuente, S. Dieguez, M. Bond, "Paleontology and stratigraphy of the Aisol Formation (Neogene), San Rafael, Mendoza" in *Cenozoic Geology of the Central Andes of Argentina* (SCS Publisher, 2011), pp. 135–154.

257. M. Pickford, B. Senut, P. Mein, J. Morales, D. Soria, M. Nieto-Diaz, J. Ward, M. Bamford, The discovery of lower and middle Miocene vertebrates at Auchas, Southern Namibia. *C. R. Acad. Sci. Paris Sér. IIa Sci. Terre Planètes* **322**, 901–906 (1996).

258. W. E. Miller, The late Pliocene las Tunas local fauna from southernmost Baja California, Mexico. *J. Paleontol.* **54**, 762–805 (1980).

259. P. M. A. Willis, R. E. Molnar, A review of the Plio-Pleistocene crocodilian genus *Pallimnarchus*. *Proc. Linn. Soc. N.S.W.* **117**, 223–242 (1997).

260. W. D. Pearse, T. J. Davies, E. M. Wolkovich, How to define, use, and interpret Pagel's λ (lambda) in ecology and evolution. *Glob. Ecol. Biogeogr.* **34**, e70012 (2025).

261. J. M. Beaulieu, D.-C. Jhwueng, C. Boettiger, B. C. O'Meara, Modeling stabilizing selection: Expanding the Ornstein–Uhlenbeck model of adaptive evolution. *Evolution* **66**, 2369–2383 (2012).

262. L. S. T. Ho, C. Ané, Intrinsic inference difficulties for trait evolution with Ornstein-Uhlenbeck models. *Methods Ecol. Evol.* **5**, 1133–1146 (2014).
